# Effect of Chalcogen-Phosphorus Substituents on Enediynes Undergoing the Bergman Cyclization

**DOI:** 10.1021/acs.inorgchem.5c02027

**Published:** 2025-08-13

**Authors:** Marcos Hendler, Travis Greene, Dominic A. Sirianni, Carol A. Parish

**Affiliations:** a Department of Chemistry, Gottwald Center for the Sciences, 6888 University of Richmond, Richmond, Virginia 23173, United States; b Department of Biochemistry & Chemistry, Westminster College, New Wilmington, Pennsylvania 16172, United States

## Abstract

We have theoretically characterized electrocyclizations of chalcogen-phosphorus-containing enediynes. We performed quantum calculations at the BS-(U)­CCSD/cc-pVDZ level to analyze the geometries and energetics of two different cyclization pathways, each consisting of the first three chalcogens (oxygen, sulfur, and selenium). The first pathway involved the cyclization of the chalcogen-phosphorus substituents followed by Bergman cyclization, while the second pathway proceeded via Bergman cyclization followed by chalcogen-phosphorus cyclization. To more accurately understand the energies of the diradicals involved in each pathway, we also performed spin-flip characterizations using UHF reference wave functions and the spin-flip formulation of the equation-of-motion coupled cluster theory with singles and doubles method. The addition of the chalcogen-phosphorus substituents to the six-membered acyclic enediyne leads to a lowering in the reaction energy of the Bergman cyclization, from +7.84 kcal/mol for (*Z*)-hexa-3-ene-1,5-diyne to +6.07, +3.71, and +3.47 kcal/mol for the oxygen, sulfur, and selenium congeners, respectively. Additionally, the formation of the doubly cyclized product is slightly unfavorable for the oxygen species (+0.70 kcal/mol) and energetically favorable for S and Se (−5.50 and −9.05 kcal/mol, respectively). The chalcogen cyclization is energetically favorable whether or not the *p*-benzyl diradical moiety is present. We also confirmed the aromaticity of these structures as well as the nature of their ground-state wave functions.

## Graphical abstract

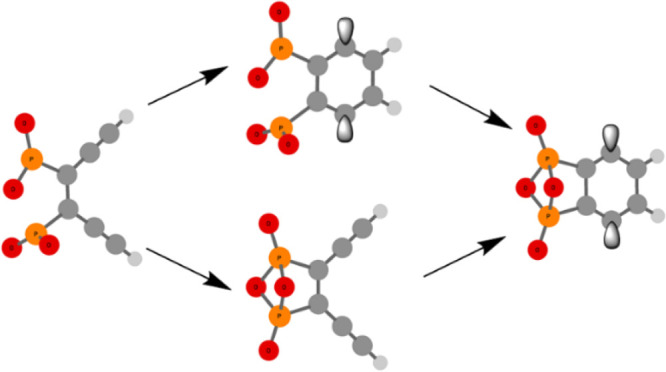

## Introduction

Enediynes undergo electrocyclization via the Bergman cyclization (Scheme 1) to form diradical products.^1,2^ The resulting diradical readily abstracts hydrogen from DNA, leading to DNA cleavage and ultimately cell death.^3–7^ The Bergman cyclization is an important mechanism in the development of cancer suppressing drugs and also has applications in organic, materials, and polymer synthesis.^8–12^

**Scheme 1:**
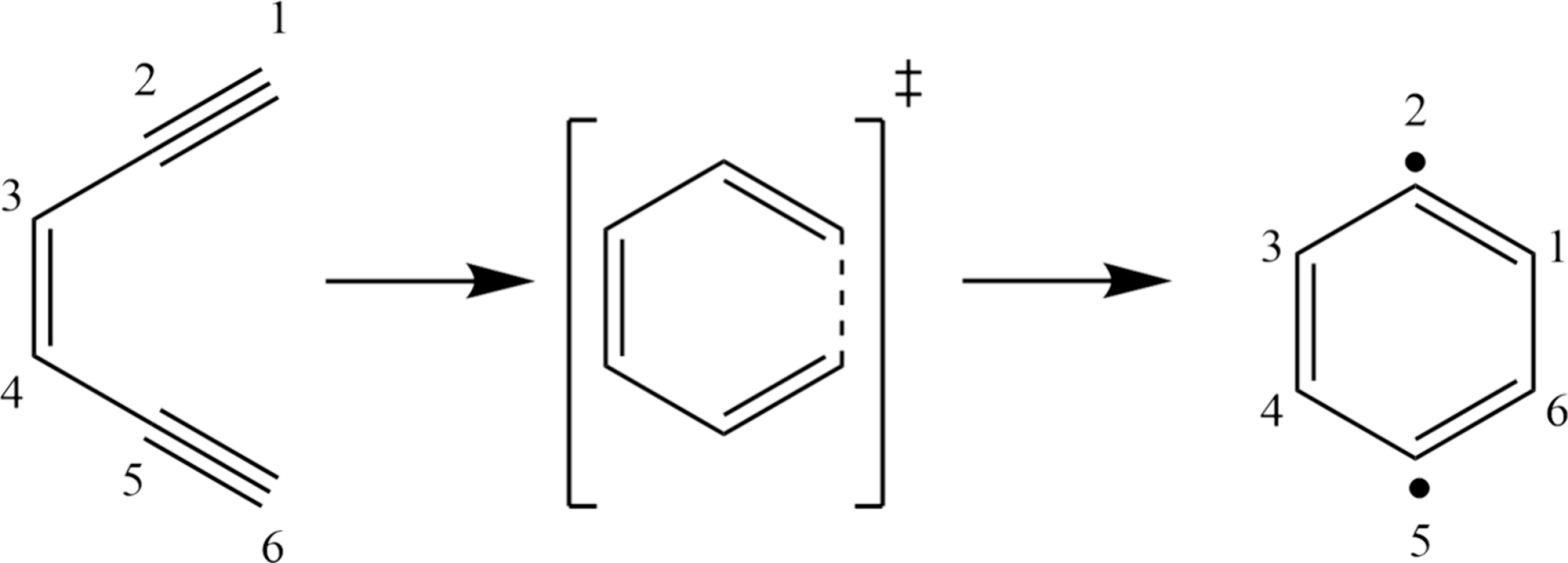
Bergman Cyclization Reaction of (*Z*)-Hex-3-ene-1,5-diyne

The Bergman cyclization of the simplest six-membered enediyne has been well-studied experimentally and computationally (Scheme 1).^1,2,13–39^ The barrier to cyclization has been experimentally determined to be 28.7 ± 0.5 kcal/mol.^1,2,7,14,25^ The reaction is endothermic, and experimental reports suggest that the Δ*E*_rxn_ is ∼8.5–13 kcal/mol.^13–15^ Previous research has shown that ene substitution alters the activation energy and allows one to tune the cyclization energetics.^40,41^

We are interested in the structure–function relationship in the Bergman reaction of enediynes, specifically how structural changes impact the cyclization energetics. In our attempts to tune the Bergman cyclization of simple enediynes, we introduced electron-donating and electron-withdrawing groups adjacent to the enediyne using electron-rich phosphate moieties as shown in Figure 1a. As part of these efforts, we discovered a secondary cyclization reaction leading to the unusual bridged product **1b**. In **1b**, the phosphorus and oxygen atoms cyclize to form a bridged five-membered ring that functionalizes the enediyne backbone. Structure **1b** has not been reported in the literature; however, there are reagents that have a similar cyclized chalcogen structural motif. The Woollins' reagent contains a selenium-substituted diphosphetane four-membered ring, while the Lawesson's reagent is substituted with sulfur (Figure 2).

**Figure 1:**
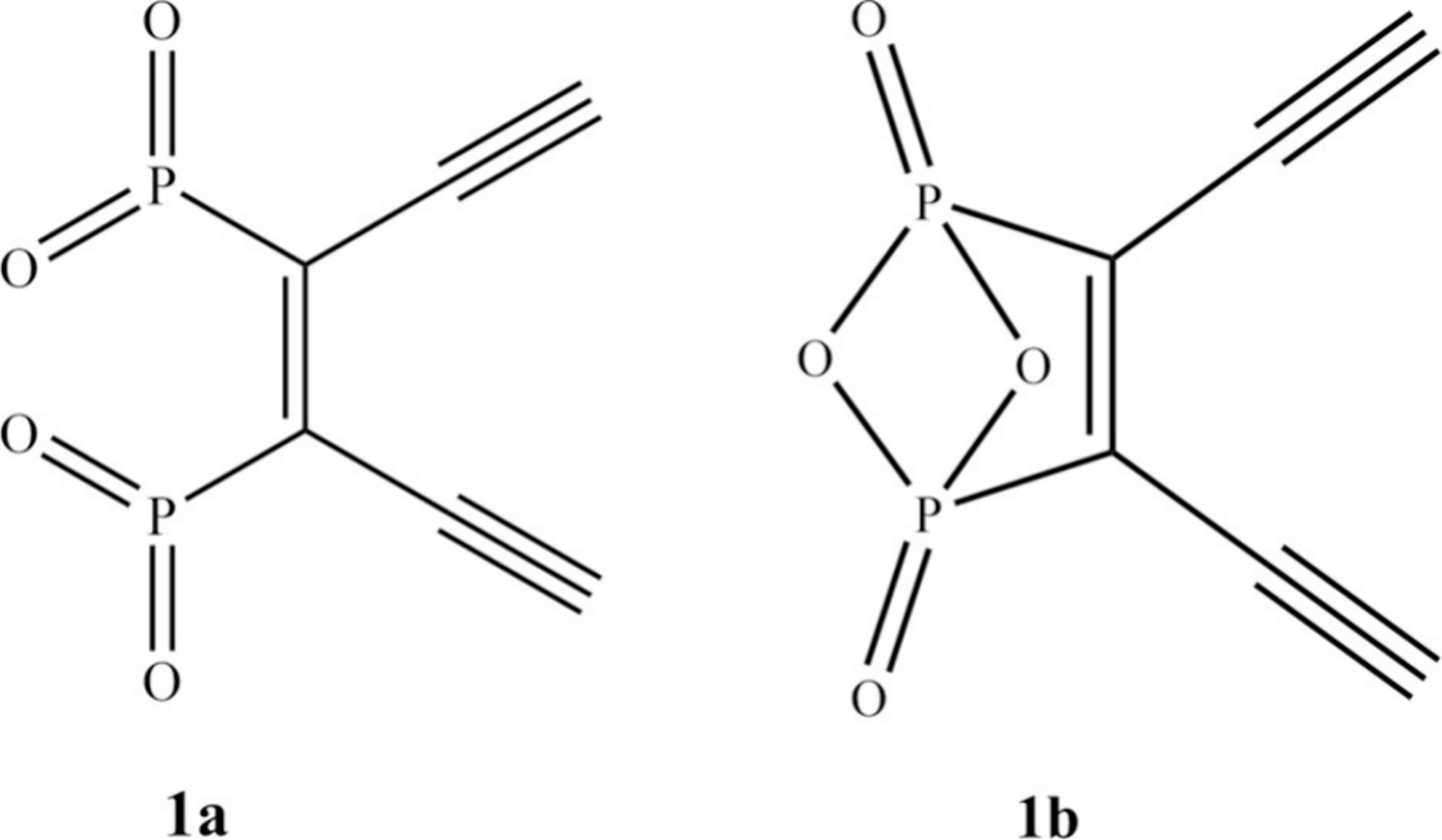
Enediyne with PO_2_ groups.

**Figure 2:**
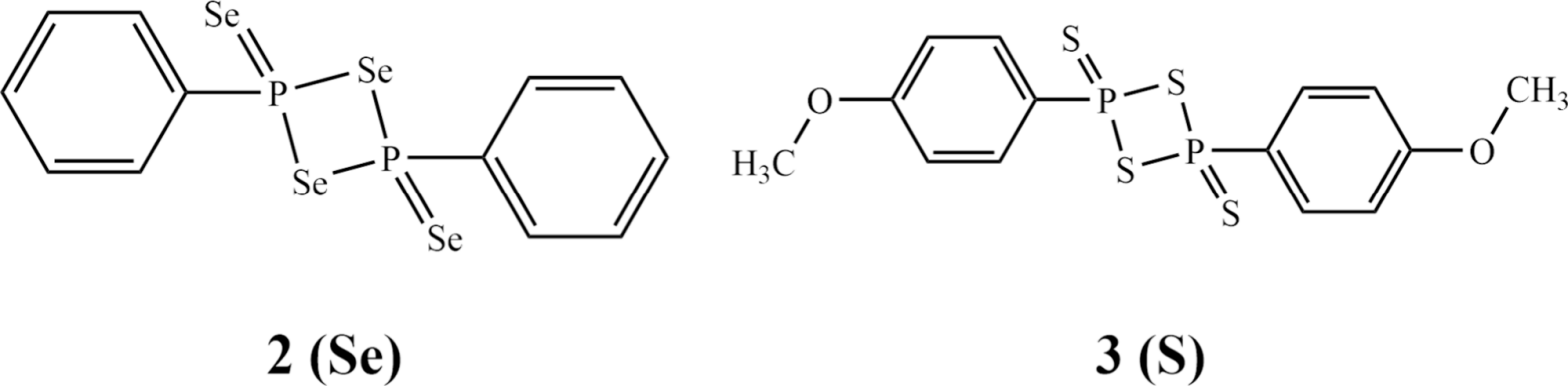
Woollins’ reagent **2** (Se) and Lawesson’s reagent **3** (S).

Woollins’ reagent **2** was first reported in 1988 and is commonly used for the synthesis of selenium-containing compounds.^42–44^ It also has applications as a highly selective reducing agent and can be used to synthesize sulfides.^45,46^ At elevated temperatures, the bicyclic form of Woollins’ reagent **2** can undergo P–Se bond scission to form two units of diselenaphosphorane, which can further react with a multitude of reagents, from sulfoxides to aryl nitriles (Scheme S1).^46,47^

Lawesson’s reagent **3** was first introduced by Lecher and colleagues in 1956 and was systematically analyzed in 1978 by Lawesson and others.^48–54^ Lawesson’s reagent is commonly utilized to perform thionation reactions, which converts carbonyl groups to thiocarbonyls (Scheme S2).^55–58^ Lawesson’s reagent can exist in equilibrium as two dithiophosphine ylides, which react with a carbonyl compound to form a thiaoxaphosphetane intermediate that produces a thiocarbonyl group as well as an oxathiophosphine ylide.^52,53,59,60^

We were intrigued by the possibility of forming novel chalcogen-containing enediynes and the impact such substitutions would have on the energetics of the Bergman cyclization. We were also interested in understanding the impact of the enediyne on the stability and energetics of the chalcogen cyclization. Given the widespread interest in utilizing the Bergman cyclization to form extended, conjugated molecules for use in pharmaceutical and polymer science, as well as in developing electro- and optical materials, we sought to better understand the congener effect of such substitution and the role such congeners play in electrocyclizations, especially those forming diradical intermediates. In what follows, we use a variety of computational techniques to examine the various cyclization pathways in enediynes containing O, S, and Se, as outlined in Scheme 2.

**Scheme 2:**
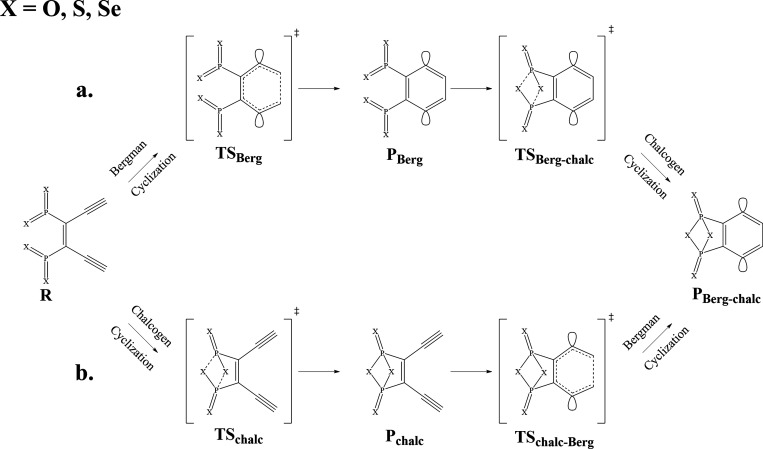
(a, b) Reaction Pathways^a^

a Note: **X** represents the different chalcogen congeners (O, S, and Se). **R** is an enediyne with two PX_2_ groups on the ene. (a; top): **TS**_**Berg**_ is the transition state of **R** undergoing the Bergman cyclization to form **P**_**Berg**_. **TS**_**Berg‑chalc**_ is the transition state of **P**_**Berg**_ undergoing the bicyclic chalcogen-phosphorus cyclization to form structure **P**_**Berg‑chalc**_. (b; bottom): **TS**_**chalc**_ is the transition state of **R** undergoing a cyclization in which the two PX_2_ groups react to form **P**_**chalc**_, an enediyne with the bicyclic chalcogen-phosphorus five-membered ring complex. **TS**_**chalc‑Berg**_ is the transition state of **P**_**chalc**_ undergoing the Bergman cyclization to form structure **P** **Berg-chalc**.

## Computational Methods

Molecular structures of all species in Scheme 2 were geometry-optimized using the coupled cluster method with single and double excitations (CCSD) along with the correlation-consistent polarized-valence double ζ (cc-pVDZ) basis set as implemented in Q-Chem 5.4.1.^61–66^ The enediyne reactants and **P_chalc_** were computed using a restricted approach, while the transition states and products were computed by using an unrestricted approach due to the potential for open-shell character. Furthermore, the two diradical structures **P**_**Berg**_ and **P**_**Berg‑chalc**_ as well as the **TS_Berg‑chalc_** transition state were calculated with broken symmetry, with a 50% mixing of the HOMO and LUMO orbitals at each optimization step. Frequency analysis was used to confirm that the optimized geometries of the reactants and products were minima on their respective potential energy surfaces and that transition states contained only a single imaginary frequency whose motion perturbed the geometry in a way that connected the reactants to the products. Absolute energies for all species can be found in Table S9. When visualizing the molecular orbitals (MOs) along the cyclization pathways, we utilized the Hartree–Fock optimized MOs that were obtained during the (BS-U)­CCSD/DZ geometry optimizations (using an unrestricted reference for transition states and broken symmetry for the transition states and diradicals). To ensure that the PX_2_ arms were oriented according to the global minimum structures, a relaxed potential energy scan of the reactant arms at the B3LYP/6-31G* level of theory was also performed.

Nucleus-independent chemical shifts (NICS) were used to assess the aromaticity of the resulting diradical products **P**_**Berg**_ and **P**_**Berg‑chalc.**_ Isotropic deshielding values were calculated using the BS-UCCSD/DZ geometries evaluated within the gauge-invariant atomic orbital (GIAO) formalism at the BS-UB3LYP/cc-pVDZ level of theory using Gaussian 16.^67–70^ Magnetic metrics of aromaticity are based on the ring current model, in which delocalized π-electrons of an aromatic molecule move around the ring in response to an applied magnetic field, thus generating an electrical current whose own magnetic field opposes the applied magnetic field.^71–74^ NICS probes were placed at the ring centroid [NICS(0)] and ±1 Å above and below the ring centroid along the *p*-benzyl moiety’s principal moment of inertia [NICS(±1)] according to the general approach developed previously.^75^

Diradical singlet products are likely to be multiconfigurational, and therefore, to fully characterize these species, we also used the spin-flip formulation of equation-of-motion coupled cluster theory with singles and doubles (EOM-SF-CCSD), along with the cc-pVDZ basis set, to characterize the diradicals **P**_**Berg**_ and **P**_**Berg‑chalc**_.^76–85^ The power of the SF method is that a multiconfigurational state (often found in a singlet diradical) can be described as a “spin-flip” operation from a single-configurational high-spin (*M*_s_ = ± 1) triplet reference state. Because EOM-SF-CCSD constructs the full, multiconfigurational excited state, computing, e.g., *T*_1_, or any other diagnostic of multiconfigurationality, is unnecessary. To perform our single-point SF calculations, we utilized the single-reference triplet wave function constructed with the unrestricted Hartree–Fock approach **|**(core)^2*n*^(σ)^α^(σ*)^α^⟩ applied to the previously optimized BS-UCCSD/DZ diradical singlet geometries.

## Results

### Cyclization Pathways Produce Stationary Points on the Potential Energy Surface

In all cases, we identified frequency-confirmed geometries corresponding to the reactant, transition state, and diradical product for each structure in the Bergman and chalcogen-phosphorus cyclization pathways for each O, S, and Se congener. As shown in Scheme 2 and Figure 3, we utilize the naming conventions listed in Table 1.

**Table 1:** Naming Convention of the Structures Used

**R**	reactants
**TS**_**Berg**_	Bergman cyclization transition state
**P**_**Berg**_	benzyne-containing diradical formed by the Bergman cyclization of the enediyne
**TS**_**Berg‑chalc**_	chalcogen cyclization transition state of the benzyne diradical
**P**_**Berg‑chalc**_	doubly cyclized chalcogen diradical
**TS**_**chalc**_	enediyne-containing chalcogen cyclization transition state
**P**_**chalc**_	chalcogen cyclized enediyne
**TS**_**chalc‑Berg**_	chalcogen cyclized Bergman cyclization transition state

**Figure 3:**
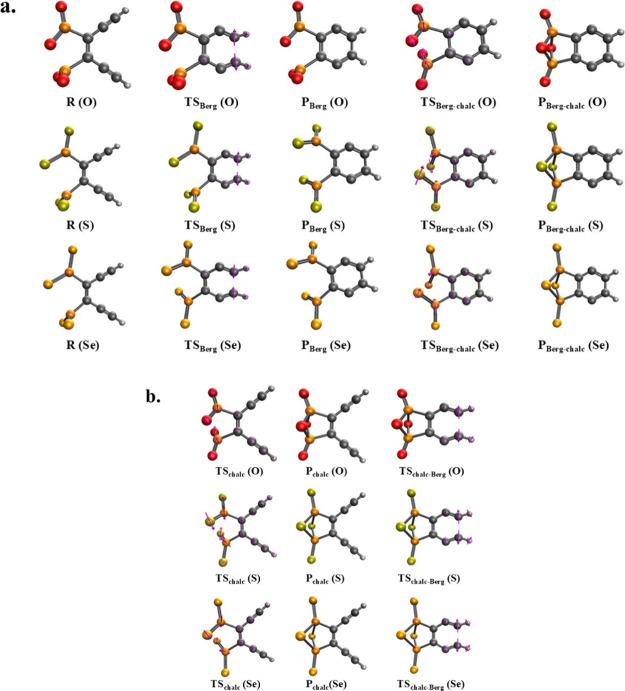
Reactant, transition state, and cyclization product structures corresponding to the reaction pathways in Scheme 2, obtained at the (BS-U)­CCSD/cc-pVDZ level of theory. (a) Optimized geometries along the Bergman cyclization pathway followed by the bicyclic chalcogen-phosphorus cyclization. (b) Optimized geometries of the bicyclic chalcogen-phosphorus cyclization followed by the Bergman cyclization. Note: Each cyclization pathway begins with structure **R** and finishes with structure **P**_**Berg‑chalc**_ as illustrated in Scheme 2.

All molecules optimize to *C*_1_ symmetric structures, except for the congeners of **P**_**chalc**_, **TS**_**chalc‑Berg**_, and **P**_**Berg‑chalc**_, which resulted in *C*_2*v*_ symmetry, and the sulfur and selenium congeners of **P**_**Berg**_, which optimized to *C*_2_ symmetry (Figure 3). For more details regarding each individual structure, see Figures S1–S8 and Tables S1–S8 in the Supporting Information.

Before characterizing the cyclization energetics for each congener, we performed conformational analysis of the PX_2_ arms in the **R** reactants. We did this to ensure that the reactant PX_2_ arms correspond to the global minimum energy conformations. The conformational analysis involved potential energy scans of the PX_2_ arms in the reactants (only), using the B3LYP/6-31G* level of theory (Figure S9a–c). The lowest energy **R** structure for all congeners is very similar on the B3LYP/6-31G* and CCSD/cc-vPDZ surfaces with RMSD superposition values of 0.04, 0.09, and 0.00 Å for the O, S, and Se conformation of **R**, respectively. These values, along with visual inspection of the superimpositions, indicate that the **R** structures on both surfaces are virtually the same. In all **R** structures, the two chalcogen-phosphorus functional groups orient perpendicular to each other (Figure S10). This conformation is stabilized by the attractive forces between the chalcogens and the phosphorus, i.e., the electronegative chalcogens (χ ranges from 2.55 to 3.44 on the Pauling scale) are more electronegative than phosphorus (χ = 2.19 on the same (Pauling) scale), causing the chalcogens to pull electron density away from the phosphorus. This results in partial positive and negative charges on the phosphorus and chalcogen atoms, respectively. The negatively charged inner chalcogens that point toward the opposing positively charged phosphorus atoms interact via dipole–dipole forces.

To better understand the Bergman and chalcogen cyclization pathways, we geometry-optimized the reactants, transition states, and cyclized products using the (BS-U)­CCSD/cc-pVDZ level of theory. We display the corresponding relative energetics in Figure 4, where all values are relative to the starting enediyne **R**. Absolute energies can be found in Table S9. Bergman cyclization energetics relative to the respective starting enediynes **R** and **P**_**chalc**_ can be found in Table S10 and Figure S11a–c. We also attempted to characterize these pathways using (BS-U)­CCSD with the triple-ζ quality basis set cc-pVTZ, but given our computational resources, we were unable to fully characterize all pathways on molecules of this size and low symmetry.

**Figure 4:**
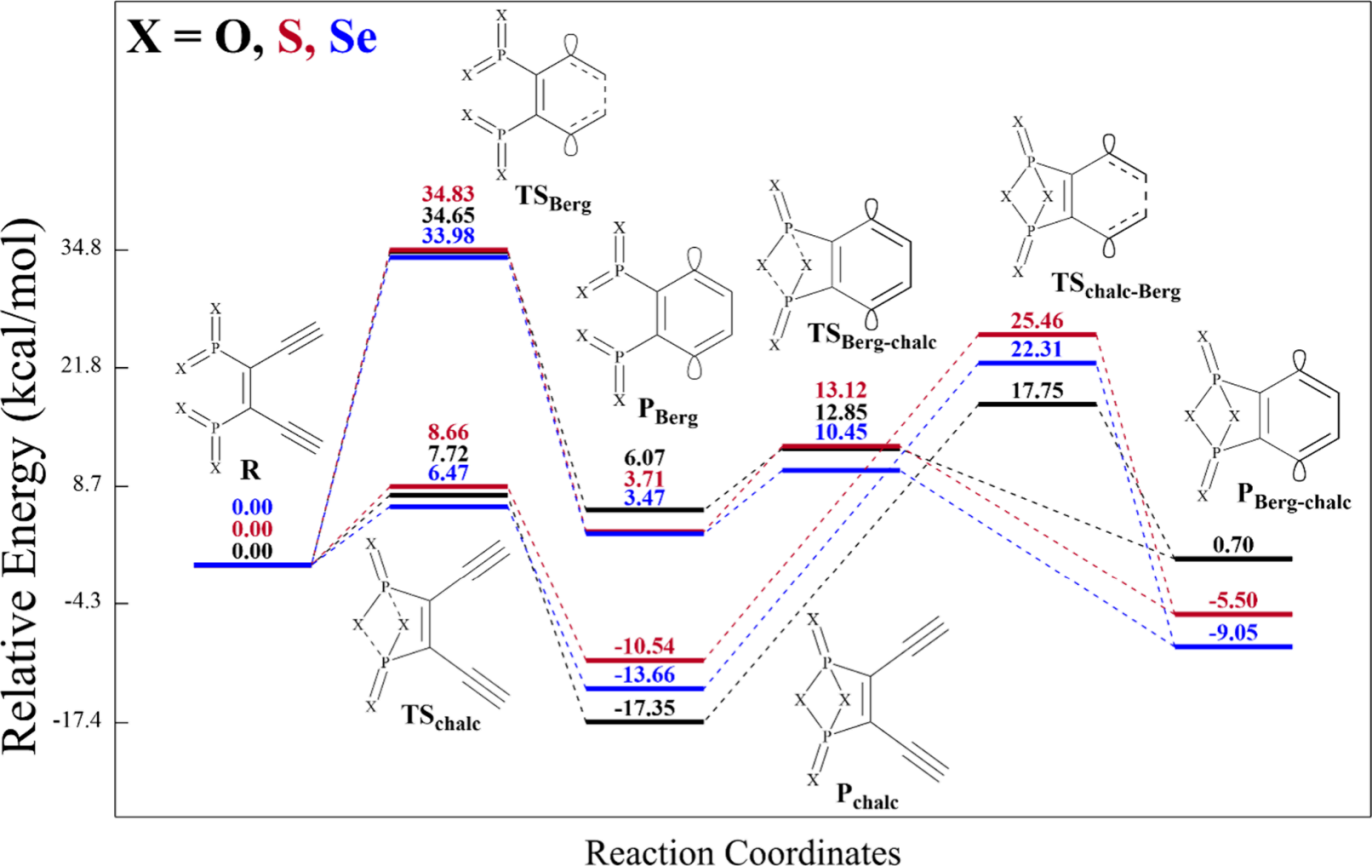
Potential energy surface for Bergman and chalcogen cyclizations, with Δ*E*_a_ and Δ*E*_rxn_ relative to structure **R** at 0 kcal/mol. Note: Pathway **R-TS**_**Berg**_ **-P**_**Berg**_ **-TS**_**Berg‑chalc**_ **-P**_**Berg‑chalc**_ starts with the Bergman cyclization while pathway **R-TS**_**chalc**_ **-P**_**chalc**_ **-TS**_**chalc‑Berg**_ **-P**_**Berg‑chalc**_ starts with the cyclization of the bicyclic chalcogen-phosphorus complex. All geometries (shown in Figure 3) and energies were determined using the BS-(U)­CCSD/cc-pVDZ level of theory.

Given that the broken-symmetry approach is an approximation to the proper characterization of a potentially multiconfigurational diradical, we also performed EOM-SF-CCSD/DZ characterization of **P**_**Berg**_ and **P**_**Berg‑chalc**_ using an unrestricted HF reference wave function. Those results are presented below the (BS-U)­CCSD/cc-pVDZ results.

### Comparing the Energetics of the Bergman and Chalcogen Cyclization Pathways

#### Pathway 2a: Bergman Cyclization followed by Chalcogen Cyclization

As we scan the respective cyclization pathways, we see that the activation barrier for the Bergman cyclization of the reactant **R** along pathway 2a (see Scheme 2a) for all congeners is within 0.85 kcal/mol of each other (Figure 4) and within 2.41 kcal/mol of the barrier at the same level of theory for the Bergman cyclization of the basic six-membered enediyene (the barrier for (*Z*)-hexa-3-ene-1,5-diyne is +36.39 kcal/mol using CCSD/cc-pVDZ (Figure S11d). Bergman cyclization from **R** to the benzyne-like diradical **P**_**Berg**_ is slightly more favorable than for the parent reaction shown in Scheme 1; the addition of the chalcogen arms lowers the reaction energy of the Bergman cyclization from +7.84 kcal/mol for (*Z*)-hexa-3-ene-1,5-diyne to +6.07 (O), +3.71 (S), and +3.47 (Se) kcal/mol, where all values were computed with (BS-U)­CCSD/cc-pVDZ. From **P**_**Berg**_, the barrier to chalcogen cyclization (**TS**_**Berg‑chalc**_) is relatively low (range = +10.45 (Se) to +13.12 (S) kcal/mol), while the formation of the doubly cyclized product (**P**_**Berg‑chalc**_) is slightly unfavorable for the oxygen species (+0.70 kcal/mol) and energetically favorable for S and Se (−5.50 and −9.05 kcal/mol, respectively).

#### Pathway 2b: Chalcogen Cyclization followed by Bergman Cyclization

If we first cyclize the chalcogen moieties and follow the energetics relative to **R**, then we see that chalcogen cyclization has a low barrier (**TS**_**chalc**_) ranging from +6.47 (Se) to +8.66 (S) kcal/mol leading to a cyclized chalcogen enediyne (**P**_**chalc**_) whose energy is lower than the reactants **R** by between −17.35 (O) and −10.54 (S) kcal/mol. Analysis of the vibrational modes in both **TS**_**chalc**_ and **TS**_**Berg‑chalc**_, as well as our inability to locate stepwise cyclization transition states on the corresponding potential energy surfaces, indicates that the two chalcogen-phosphorus arms cyclize in a concerted fashion. The resulting chalcogen-phosphorus enediyne (**P**_**chalc**_) can undergo Bergman cyclization, with an activation barrier similar to the first step of pathway 2a. This first set of activation barriers (**TS**_**chalc‑Berg**_) for pathway 2b ranges from +17.75 (O) to +25.46 (S) kcal/mol relative to **R** (Figure 4) and +35.09 (O) to +36.00 (S) kcal/mol relative to **P**_**chalc**_ (Figure S11b).

If we compare the effect of an acyclic vs cyclic chalcogen moiety on the overall energetics of the Bergman cyclization, then we see that both acyclic and cyclic chalcogen-containing enediynes produce similar activation barriers (Table S10, Figure 4, and Figure S11a,b); however, the overall reaction energies depend on the nature of the chalcogen. For instance, we see that an enediyne containing a cyclized chalcogen (**P**_**chalc**_) produces a diradical (**P**_**Berg‑chalc**_) that is +18.04 (O), +5.04 (S), and +4.61 (Se) kcal/mol higher in energy (Figure S11b), while an enediyne containing an acyclic chalcogen (**R**) produces a diradical (**P**_**Berg**_) that is destabilized by only +6.07 (O), +3.71 (S), and +3.47 (Se) kcal/mol (Figure 4 and Figure S11a). This destabilization is more than the corresponding destabilization of the diradical relative to the simple enediyne in the parent reaction, especially for the congener containing O. Such destabilization in the parent reaction is +7.84 at the BS-UCCSD/cc-pVDZ level of theory (Figure S11d).

If we compare the relative energetic cost of forming a diradical product (**P**_**Berg**_ or **P**_**Berg‑chalc**_) either with the uncyclized (Figure S11a) or cyclized chalcogens (Figure S11b), then we see that the presence of the cyclized chalcogen causes an increase in diradical energy for all chalcogens, especially so for O where the destabilization is +11.97 kcal/mol (as opposed to the destabilization in the larger chalcogens of +1.33 (S) and +1.14 (Se) kcal/mol). (These numbers are obtained by taking the difference between the **P**_**Berg‑chalc**_ energies in Figure S11a,b.) Because the formation of the new C—C bond and diradical centers of both **P**_**Berg**_ (step 1 of pathway 2a) and **P**_**Berg‑chalc**_ (step 2 of pathway 2b) involves orbital transformations in the plane of the forming ring, this keeps electron density away from the chalcogenic groups. We hypothesize that this destabilization arises from the fact that the cyclized chalcogen moiety of **P**_**chalc**_ will be more strongly electron-withdrawing (thanks to bond dipole cooperativity) relative to the separate, more flexible, acyclic arms of **R**.

Interestingly, the presence of the *p*-benzyne-like diradical affects the barrier to chalcogen cyclization. For instance, the barriers for chalcogen cyclization shown (**TS**_**chalc**_) in Figure 4 are +6.47 (Se), +7.72 (O), and +8.66 (S) kcal/mol in the presence of the enediyne while Figure S11c shows barriers (**TS**_**Berg‑chalc**_) of +6.98 (Se), +6.79 (O), and +9.42 (S) kcal/mol in the presence of the diradical. These changes produce a correspondingly comparable decrease in barrier height for O (−0.93 kcal/mol) and a slight increase for S (+0.76 kcal/mol) and Se (+0.51 kcal/mol) when the diradical is present. This small increase in the S and Se barriers to chalcogen cyclization could be due to electron repulsion between these larger atoms and the benzyne radicals. From the frontier molecular orbitals visualized in Figures 5 and 6 below, the presence of the *p*-benzyl moiety in **P**_**Berg**_ seems to inject a good deal of electron density onto the chalcogen arms that is not present in the fully acyclic reactant **R**, making the chalcogens less easily able to maneuver relative to one another thanks to steric hindrance.

**Figure 5:**
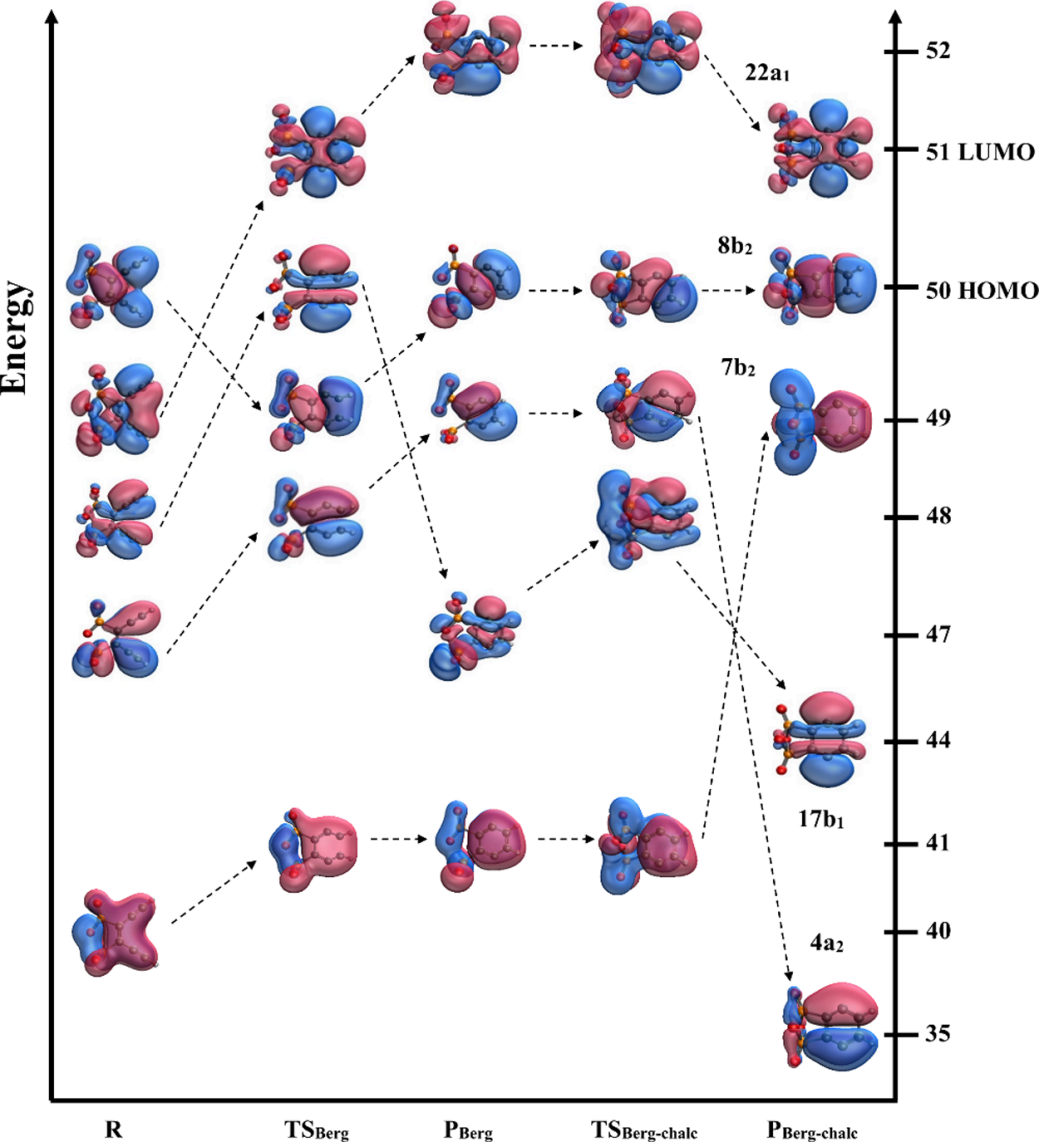
Transformation of frontier molecular orbitals along the **R-TS**_**Berg**_ **-P**_**Berg**_ **-TS**_**Berg‑chalc**_ **-P**_**Berg‑chalc**_ reaction pathway. Orbitals shown are UHF optimized MOs that were obtained during the UCCSD/cc-pVDZ geometry optimization. In the figure, the orbitals are superimposed on those optimized geometries and are ordered according to UHF energies. For the transition states and diradical intermediates, we utilized broken-symmetry guess wave functions obtained by a 50:50 mixing of the HOMO and LUMO orbitals. On the right-hand side *y*-axis, the orbital numbers are included along with labels for the highest occupied and lowest unoccupied orbitals. It was not possible to list energy values along the left-hand side *y*-axis as the UHF orbital energies are relative to each structure and not comparable across structures. For ease of visualization, only alpha orbitals are shown.

**Figure 6:**
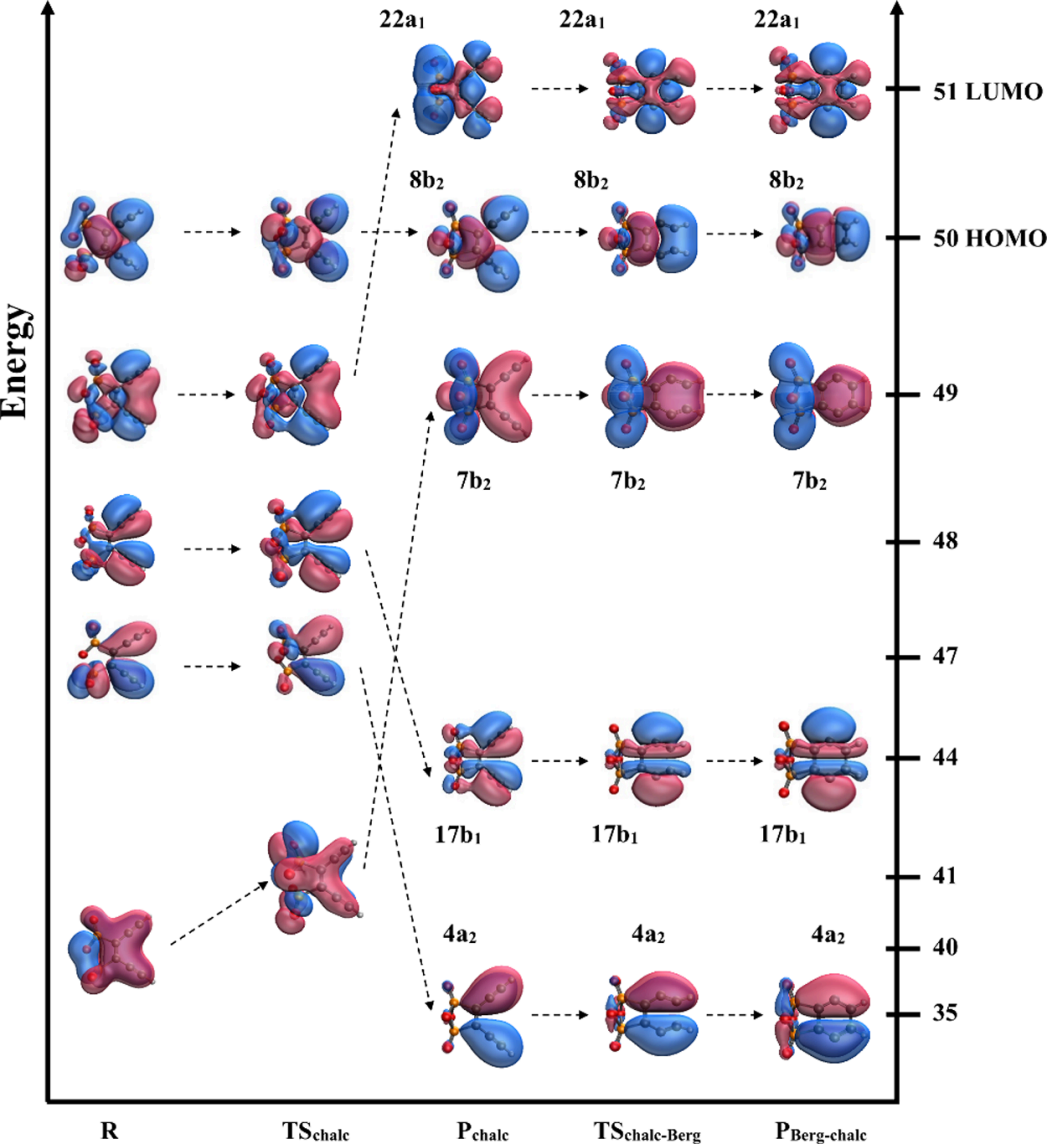
Transformation of frontier molecular orbitals along the **R-TS**_**chalc**_ **-P**_**chalc**_ **-TS**_**chalc‑Berg**_ **-P**_**Berg‑chalc**_ reaction pathway. Orbitals shown are UHF optimized MOs that were obtained during UCCSD/cc-pVDZ geometry optimization. In the figure, the orbitals are superimposed on those optimized geometries and ordered according to the UHF energies. For the transition states and diradical intermediates, we utilized broken-symmetry guess wave functions obtained by a 50:50 mixing of the HOMO and LUMO orbitals. On the right-hand side *y*-axis, the orbital numbers are included along with labels for the highest occupied and lowest unoccupied orbitals. It was not possible to list energy values along the left-hand side *y*-axis, as the UHF orbital energies are relative to each structure and not comparable across structures. For ease of visualization, only alpha orbitals are shown.

Chalcogen cyclization is energetically favorable whether the *p*-benzyl diradical moiety is present. For instance, Figure S11a shows that the chalcogen cyclization of the enediyne is favorable by −10.54 to −17.35 kcal/mol, whereas chalcogen cyclization in the presence of the diradical is favorable by −5.37 to −12.52 kcal/mol (Figure S11c). Again, we see differential effects depending on the nature of the chalcogen; in particular, the O congener changes from being the most favorable in the presence of the enediyne (−17.35 kcal/mol) to being the least favorable in the presence of the diradical (−5.37 kcal/mol) (Figure S11a,c). This is again most likely an artifact of the differences in the electronegativity of these chalcogens. The tricyclic chalcogen moiety will be most strongly electron-withdrawing in the oxygen congener (χ = 3.44) followed by sulfur (χ = 2.58) and selenium (χ = 2.55), inversely related to the stability of the congeners of **P**_**Berg‑chalc**_ (Figure 4) thanks to the requirement for electron density to be sequestered into the new C–C bond and diradical lobes of the *p*-benzyl moiety. For comparison, the chalcogen cyclization of the simple (*Z*)-1,2-bis­(dioxo/thio/seleno)­phosphoranyl)­ethene has a relatively low cyclization barrier (ranging from +9.69 kcal/mol for O to +13.15 kcal/mol for S) and produces a cyclized chalcogen product that is lower in energy than the reactant by −14.61 kcal/mol for O and −4.85 kcal/mol for S (Figure S11e).

The activation barrier for chalcogen cyclization (**TS**_**chalc**_ and **TS**_**Berg‑chalc**_) is highest for the sulfur congeners (Figure S11a,c). This may be due to the hypervalency of sulfur and selenium.^86^ In hypervalent situations, selenium’s larger size relative to sulfur allows selenium to adopt geometries that reduce unfavorable steric interactions. This leads to the selenium congeners having a lower activation barrier and a more stable product than sulfur. Oxygen has the lowest activation barrier (**TS**_**Berg‑chalc**_), and this may be due to the Bergman diradical drawing electron density away from the chalcogen-phosphorus cyclization. On the other hand, oxygen has a higher activation barrier than selenium for structure **TS**_**chalc**_ since oxygen is the smallest chalcogen. This leads to shorter phosphorus-chalcogen bonds in the oxygen congeners and a buildup of electron density in the interior of the ring, which destabilizes the transition state, but not as much as the effects of the hypervalency in sulfur.

Originally, we hypothesized that **P**_**Berg‑chalc**_ would be unstable and that the formation of the diradical (i.e., **P**_**chalc**_ → **TS**_**chalc‑Berg**_ → **P**_**Berg‑chalc**_) would lead to a rupture of the tricyclic chalcogen moiety, given the high density of electrons in a such a small molecular space, along with significant ring strain. However, our results indicate that **P**_**Berg‑chalc**_ is a stable structure, and this is in keeping with previous calculations on the Woollins’ and Lawesson’s reagents indicating that energetic costs for ring rupture are +19.6 and +24.5 kcal/mol, respectively.^54,87^

To summarize the mechanistic details provided above, we can say that chalcogen cyclization is kinetically and enthalpically facile. Chalcogen substitution at the ene locations of the enediyne, either acyclic or cyclic, plays a small role in changing the Bergman cyclization energetics; however, the formation of the doubly cyclized diradical **P**_**Berg‑chalc**_ is energetically favorable relative to **R** when the enediyne contains S and Se. Of course, even small changes in barrier heights play a significant role in reaction rates and the lowering of the Bergman barrier seen in the acyclic chalcogen-containing enediynes by between −1.5 and −2.4 kcal/mol would be expected to increase the rate of cyclization by at least 12-fold according to the straightforward Arrhenius description of this unimolecular elementary step in these reaction pathways. It should be noted that, while it was not possible to obtain higher quality geometries for molecules of this size and electronic complexity, the BS-(U) CCSD/DZ approach that we have taken does produce diradical singlet-state intermediates that are spin-contaminated by low lying triplets. This mixing of spin states likely leads to relative energies for **P**_**Berg**_, **TS**_**Berg‑chalc**_, and **P**_**Berg‑chalc**_ that are larger than those if the mixing did not occur.

### Analyzing Molecular Orbital Transformation along Each Cyclization Pathway

We utilized molecular orbital (MO) symmetry and visualization to better understand how the σ, σ*, and occupied π MOs transform along the two different cyclization pathways to form the diradicals **P**_**Berg**_ and **P**_**Berg‑chalc**_ (Figures 5 and 6). It is important to note that while the orbital shapes are likely mostly correct at the UHF/cc-pVDZ level (and with the broken-symmetry approach applied to all species containing a diradical moiety), the energetic ordering is qualitative. It is also the case that, due to the complexity of these molecules, there are multiple σ, σ*, and occupied π MOs; in constructing Figures 5 and 6, we focused on those frontier orbitals closest to the HOMO–LUMO gap.

When the Bergman cyclization occurs before chalcogen cyclization (pathway 2a), we see significant shuffling of the orbitals along the cyclization pathway (Figure 5). In **R**, we can see the beginnings of the σ (MO 49) and σ* (MO 48) orbitals in the occupied manifold, and as we progress to **TS**_**Berg**_ and then to **P**_**Berg**_, we can see σ* dropping lower, while σ moves higher in the manifold. This should not be surprising, as the stabilization of σ* relative to σ in diradical species like **P**_**Berg**_ (and its “parent,” the unsubstituted *p*-benzyne) is a well-known effect of the interaction of radical electrons mediated by through-bond coupling originally described by Hoffmann.^88,89^ As the reaction proceeds through the second transition state (associated with chalcogen cyclization, **TS**_**Berg‑chalc**_), the lowest occupied π orbital in **TS**_**Berg‑chalc**_ is destabilized in **P**_**Berg‑chalc**_ (MO 41 in **TS**_**Berg‑chalc**_ becomes MO 49 in **P**_**Berg‑chalc**_) while the second lowest π orbital in **TS**_**Berg‑chalc**_ is significantly stabilized in **P**_**Berg‑chalc**_ (MO 49 in **TS**_**Berg‑chalc**_ becomes MO 35 in **P**_**Berg‑chalc**_). This stabilization is due to the incorporation of a significant amount of the chalcogen ring electron density into this π orbital, as can be seen in MO 35 of **P**_**Berg‑chalc**_.

For pathway 2b, when the chalcogen cyclization precedes the Bergman cyclization, we see less orbital shuffling in the energetic manifold (Figure 6). Again, we see the beginnings of the σ (MO 49) and σ* (MO 48) orbitals in the occupied manifold of **R**, and a destabilization of the lowest occupied π orbital as the second π orbital moves lower due to the sharing of electron density with part of the chalcogen moiety. Interestingly, the orbital that will become the lowest lying π MO in **P**_**Berg‑chalc**_ (MO 35) develops sooner in the reaction progression, i.e. this orbital develops in **P**_**chalc**_ (MO 35) rather than only in **P**_**Berg‑chalc**_ as shown for pathway 2a (Figure 5).

Regardless of the pathway 2a vs 2b, in the final product **P**_**Berg‑chalc**_, we see an approximate energetic ordering of π_1_ < σ* < π_2_ < π_3_ < σ. This MO ordering is confirmed in our results at the EOM-SF-CCSD level of theory (*vide infra*). Through-bond coupling occurs in both diradical species, as evidenced by the σ* orbital lying lower in energy than the σ orbital.^88,89^

### Assessing Aromaticity in the **P**_**Berg**_ and **P**_**Berg‑chalc**_ Diradicals

The driving force for the canonical Bergman cyclization of (*Z*)-hexa-3-ene-1,4-diyne is the aromatization of the resulting *p*-benzyne diradical.^31,75^ Aromaticity has also shown to be a major contributor to the Bergman-type cyclization of ionic (pseudo)­enediynes.^75^ While aromatization is also a likely contributor driving Bergman cyclization in the disubstituted enediynes explored here, it is unknown to what degree these strongly electron-withdrawing PX_2_ groups will perturb the aromaticity of **P**_**Berg**_ and **P**_**Berg‑chalc**_. To assess the aromaticity of these diradical species, we computed isotropic nucleus-independent chemical shifts (NICS) within the gauge-invariant atomic orbital (GIAO) formalism at the BS-UB3LYP/cc-pVDZ level of theory using Gaussian 16 (Table 2).^90^ At their BS-UCCSD/cc-pVDZ optimized geometries, both **P**_**Berg**_ and **P**_**Berg‑chalc**_ are significantly aromatic regardless of chalcogen, in a fashion similar to *para*-benzyne. All of these diradicals are markedly more aromatic than benzene, whose isotropic deshieldings were also computed with NICS at the B3LYP/cc-pVDZ level of theory.

**Table 2:** Isotropic Deshieldings (ppm) for Each Diradical Structure, Computed via Nucleus-Independent Chemical Shifts (NICS) Determined at the BS-UB3LYP/cc-PVDZ Level of Theory with CCSD/DZ Geometries^a^

		NICS index		
species	chalcogen	–1	0	+1
**P**_**Berg**_	oxygen	–13.0	–16.7	–13.0
	sulfur	–12.0	–15.8	–12.0
	selenium	–11.7	–15.7	–11.7
**P**_**Berg‑chalc**_	oxygen	–13.4	–18.5	–13.4
	sulfur	–16.2	–25.2	–16.2
	selenium	–15.3	–25.1	–15.3
benzene	NA	–11.0	–8.9	–11.0
*p*-benzyne	NA	–13.4	–16.9	–13.4

a NICS probes were placed at the ring centroid [NICS(0)] and ±1 Å above and below the molecule’s principal moment of inertia [NICS(±1)]. For reference, the NICS­(−1, 0, +1) values for benzene were computed by using the same approach at the B3LYP/cc-pVDZ level of theory.

Between these two diradical species, the increase in aromaticity observed upon chalcogen cyclization (**P**_**Berg**_ → **P**_**Berg‑chalc**_) likely arises from an increased global aromaticity for the tricyclic **P**_**Berg‑chalc**_ relative to the monocyclic ring current of **P**_**Berg**_, consistent with previous observations by some of us for bicyclic aromatic molecules.^75^ This finding is also noteworthy, as it absolves a lack of aromaticity from being the cause of the slight destabilization of **P**_**Berg‑chalc**_ relative to the **P**_**chalc**_ intermediate of Scheme 2b. Interestingly, however, this increase in aromaticity does not fully justify the trend of increasing stability of O → S → Se for **P**_**Berg‑chalc**_, as the sulfur congener is slightly more aromatic than its selenium counterpart. This further illuminates the delicate interplay between the electron-withdrawing strength of these cyclic vs acyclic groups and the electronic rearrangements necessary for new bond formation.

### Quantitative Characterization of the **P**_**Berg**_ and **P**_**Berg‑chalc**_ Diradicals Using the Spin-Flip EOM-CCSD Method

We also performed spin-flip (EOM-SF-CCSD/cc-pVDZ) calculations on the **P**_**Berg**_ and **P**_**Berg‑chalc**_ diradical species using the BS-UCCSD/cc-pVDZ geometries in order to more accurately characterize the diradical energetics as well as the multiconfigurational nature of their ground-state wave functions (Table 3). The discussion below focuses on the leading determinants for these multiconfigurational diradicals. Full details regarding the wave functions can be found in Tables S11a–c and S12a–c.

**Table 3:** Spin-Flip Energetics for All Congeners of Structures **P**_**Berg**_ and **P**_**Berg‑chalc**_ ^d^

**P**_**Berg**_ **(chalcogen)**	state^a^	Δ*E*_ST_ (eV)^b^	⟨*S* ^*2*^⟩^c^	leading determinants in a spin-flipped excited singlet state^d^ ^,^ ^e^
oxygen	**X^1^ *A***	–0.2365	0.454	47% × |(core)^98^(1σ*)^αβ^⟩
sulfur	**X^1^ *A***	–0.0940	0.011	38% × |(core)^130^(1σ*)^αβ^⟩
selenium	**X^1^ *A***	–0.0819	0.102	43% × |(core)^202^(1σ*)^α^(2σ*)^β^⟩
**P**_**Berg‑chalc**_ **(chalcogen)**				
oxygen	**X^1^ *A*_1_**	–0.4125	0.011	59% × |(core)^98^(1σ*)^αβ^⟩
sulfur	**X^1^ *A*_1_**	–0.1818	0.010	20% × |(core)^130^(2σ*)^αβ^⟩ + ...
selenium	**X^1^ *A*_1_**	–0.1581	0.010	36% × |(core)^202^(2σ*)^αβ^⟩ + ...

a Schönflies symbols for each electronic state utilize the Mulliken convention for orienting symmetry axes in the *C*_2*v*_ point group.

b Vertical singlet–triplet excitation energies were calculated as Δ*E*_ST_ = *E*_Excited Singlet_ – *E*_Triplet Reference_.

c Only excitation states with an *⟨S* ^2^⟩ value within ±0.1 of a spin eigenvalue (singlet = 0, triplet = 2) were analyzed. Exceptions are made for spin-contaminated excited states that can be readily explained and complex orbital transitions.

d Reference and spin-flipped excited states were prepared at the UHF/cc-pVDZ and EOM-SF-CCSD/cc-pVDZ levels of theory, respectively, by using *C*_1_ structures for the oxygen congener of **P**_**Berg**_, *C*_2_ structures for the sulfur and selenium congeners of **P**_**Berg**_, and *C*_2*v*_ structures for **P**_**Berg‑chalc**_ optimized at the CCSD/cc-pVDZ level of theory. The reference wave function was constructed at the UHF/cc-pVDZ level of theory to be |(core)^2*n*^(σ)^α^(σ*)^α^⟩. (core)^2*n*^ denotes the first *n* doubly occupied, lower energy molecular orbitals.

e Relative weights for each Slater determinant were computed as the square of the spin-flip excitation amplitudes. Only dominant weights are shown in the table. To see the complete list of weights, see Tables S11a–c and S12a–c in the Supporting Information.

We find that the diradicals **P**_**Berg**_ and **P**_**Berg‑chslc**_ are multiconfigurational ground-state singlets for all congeners. This can be seen as negative excitation energies in Table 3 as the spin-flip procedure generates excitations from the single-reference triplet state, prepared at the UHF/cc-pVDZ level; a negative excitation energy indicates a singlet state that lies lower in energy than the high-spin triplet reference. The most significant contributors to the singlet ground-state wave function of the oxygen congener of structure **P**_**Berg**_ are |(σ*)^αβ^⟩ and |(σ)^αβ^⟩, indicating that the ground-state wave function is a two-configurational, closed-shell singlet (TCS) state in analogy to the ground state of *p*-benzyne.^31^ The negative excitation energy indicates that the singlet lies −0.237 eV below the single-reference triplet. Unfortunately, due to mixing of sigma- and π-type molecular orbitals facilitated by the nonplanar, low-symmetry molecular structure of the oxygen congener (*C*_1_), this ground-state wave function experiences a moderate degree of spin contamination (⟨*S* ^2^⟩ = 0.454), so the absolute magnitude of the energy of this electronic state should be treated with caution. The selenium congener of **P**_**Berg**_ is also slightly spin-contaminated (⟨*S* ^2^⟩ = 0.102), but the major distinguishing feature for this species is that the ground-state wave function is an admixture of an open-shell singlet (OSS) and a TCS, with the OSS actually being the leading contributor to the total wave function. Finally, the sulfur congener has a well-behaved TCS ground state that is fairly spin-pure (⟨*S* ^2^⟩ = 0.011). Interestingly, the singlet–triplet splitting for both sulfur and selenium congeners is small, with Δ*E*_ST_ = −0.094 and −0.082 eV, respectively.

For **P**_**Berg‑chalc**_, the ground-state wave function for the oxygen congener is also dominated by a TCS, lying −0.4125 eV below the high-spin triplet reference. Unlike the oxygen congener of **P**_**Berg**_, however, this ground-state wave function is fairly spin-pure with ⟨*S* ^2^⟩ = 0.011. While the sulfur and selenium congeners of **P**_**Berg‑chalc**_ are also similarly spin-pure (⟨*S* ^2^⟩ = 0.010 for each), the ground-state wave functions for those species are significantly more multiconfigurational, due to the fact that multiple σ-, σ*-, and π-type MOs are present for S and Se congeners. Specifically, the total ground-state wave function for the sulfur congener of this species is an admixture of at least six distinct singlet states (3 TCSs and 3 OSSs), and the total ground-state wave function for the selenium congener is an admixture of at least five distinct TCS states. Despite this complexity, however, the leading configuration in each wave function is still |(σ*)^αβ^⟩, which can be interpreted as an indication that they are still primarily a TCS state.

## Conclusions

We utilized the CCSD ansatz in combination with the cc-pVDZ basis set to characterize cyclization reactions for a series of chalcogen-phosphorus enediyne congeners containing O, S, and Se. We find that the addition of the chalcogen-phosphorus arms to the ene backbone has little effect on the energetic barrier of the Bergman cyclization (Figure 4 and Figure S11a). The presence of the diradical benzyne does cause the barrier for chalcogen-phosphorus cyclization to be dependent on the nature of the congener (Figure S11c). On the other hand, the reaction energy of both pathways is affected by the addition of the chalcogen-phosphorus arms as well as the nature of the congener. For instance, in the presence of the cyclized chalcogen-phosphorus moiety, the reaction energy for the Bergman cyclization is increased relative to the reaction energy for (*Z*)-hexa-3-ene-1,5-diyne and significantly greater for oxygen-containing systems (Figure S11b). The reaction energy for chalcogen-phosphorus cyclization is also higher in the presence of the benzyne diradical (comparing the lower PES in Figure S11a to Figure S11b). Overall, we find that chalcogen-phosphorus cyclization is both kinetically and enthalpically more favorable than Bergman cyclization.

Our spin-flip characterization of the diradicals suggests that both **P**_**Berg**_ and **P**_**Berg‑chalc**_ are multiconfigurational ground-state singlets. Sulfur and selenium congeners are more multiconfigurational due to the presence of multiple σ- and π-type orbitals in their occupied orbital subspaces. We do see some spin contamination in these results owing to the low symmetry of these complex structures that allow σ and π orbital mixing in accordance with El Sayed’s rule.^91^

## Supplementary Material





## Notes

Optimized geometries, energies, and nucleus-independent chemical shifts (PDF)
All computed molecular structures as *XYZ* Cartesian coordinates, absolute energies, and lowest vibrational frequencies in a format for convenient visualization (XYZ)
