# Supplementary material for: Effect of Chalcogen-Phosphorus Substituents on Enediynes Undergoing the Bergman Cyclization
Source: Inorg Chem. 2025 Aug 13;64(33):16865–76. doi: 10.1021/acs.inorgchem.5c02027 (PMC12381858; doi:10.1021/acs.inorgchem.5c02027)
Supplement: Supplementary file 1 [file ic5c02027_si_001.pdf]

The Effect of Chalcogen-Phosphorus Substituents on Enediynes Undergoing the Bergman Cyclization

Marcos Hendler,[a] Travis Greene, [a] Dominic A. Sirianni[b] and Carol Parish*[a]

(a) *Department of Chemistry, Gottwald Center for the Sciences, University of Richmond, Richmond, VA 23173*
(b) *Department of Natural Sciences & Mathematics, Daemen University, Amherst, NY 14226*

Keywords: Bergman cyclization, chalcogen-phosphorus cyclization, molecular orbital transformation, spin flip characterization of diradicals

* Corresponding Author cparish@richmond.edu

Supplemental Information

Table of Contents

Reactions facilitated by the Woollins reagent: **Scheme S1**

Reactions facilitated by the Lawesson reagent: **Scheme S2**

Chalcogen-Phosphorus Structures: **Figures S1 – 8**

Bond/Angle Tables of Structures: **Tables S1 – 8**

Potential Energy Scans of Congeners of R: **Figure S9**

Lowest Energy Congeners of R: **Figure S10**

Absolute energies and S^2 values for all species: **Table S9**

Relative Energies of Individual Reactions: **Table S10**

Potential Energy Diagrams of Individual Reactions: **Figure S11**

Wavefunction of Excited States for Congeners of P_{Berg}: **Tables S11a–c**

Wavefunction of Excited States for Congeners of $P_{Berg-chalc}$: **Tables S12a–c**

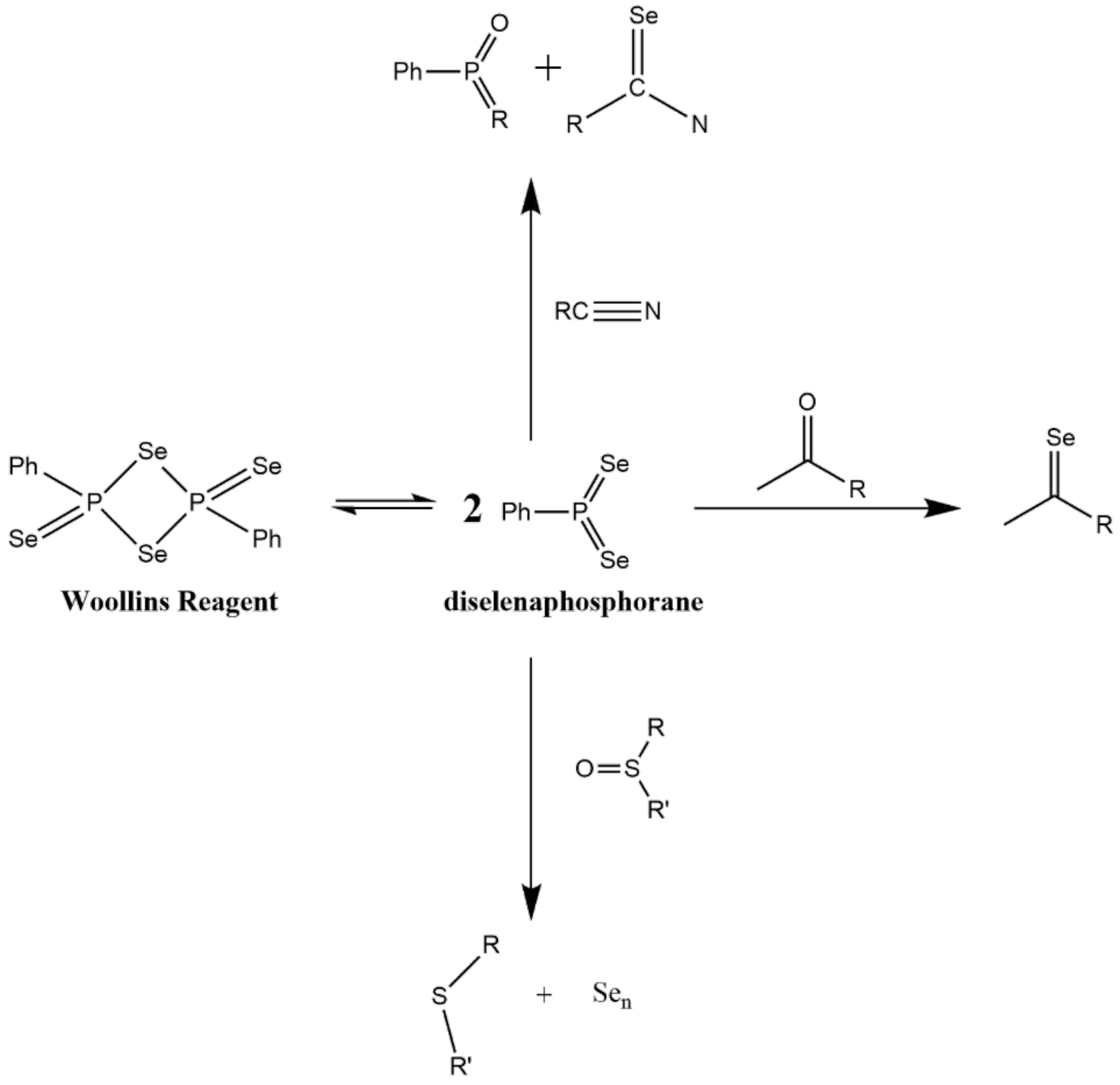

Scheme S1. Reactions facilitated by the Woollins reagent.

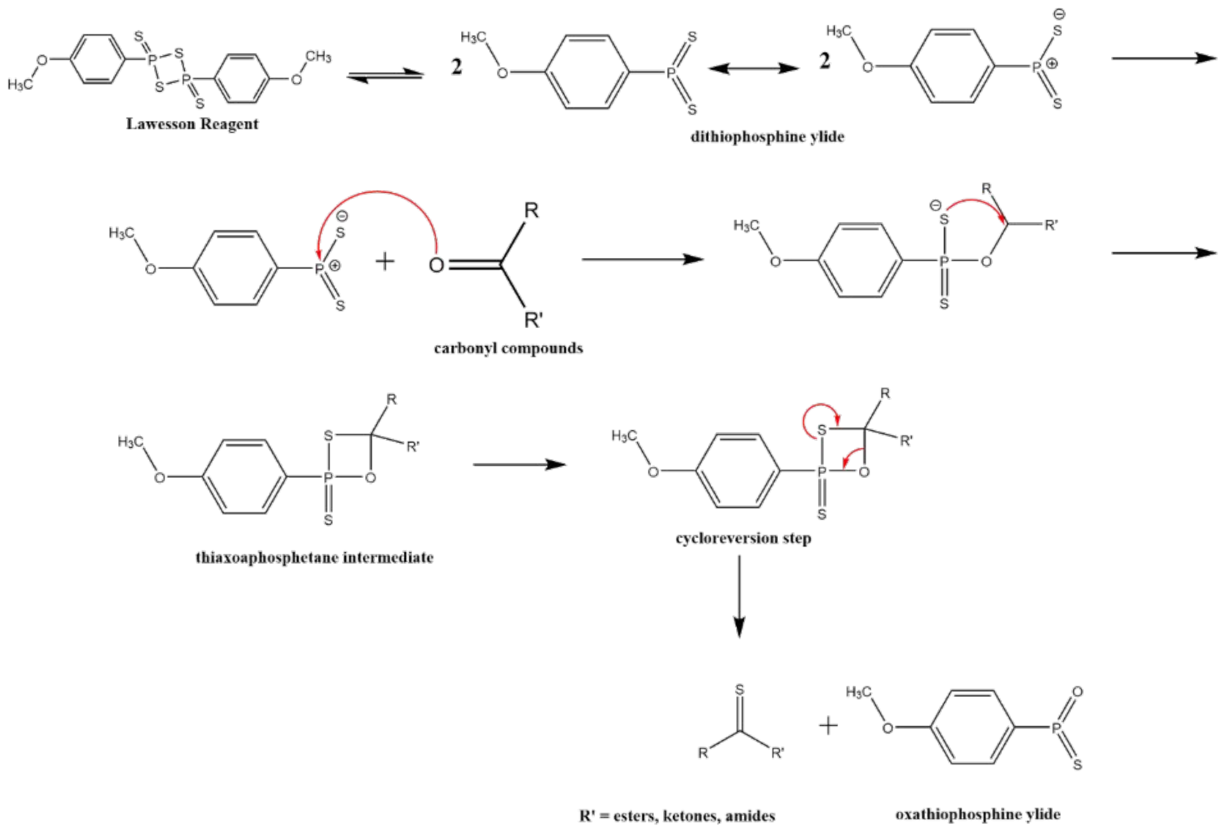

Scheme S2. Reactions facilitated by the Lawesson reagent.

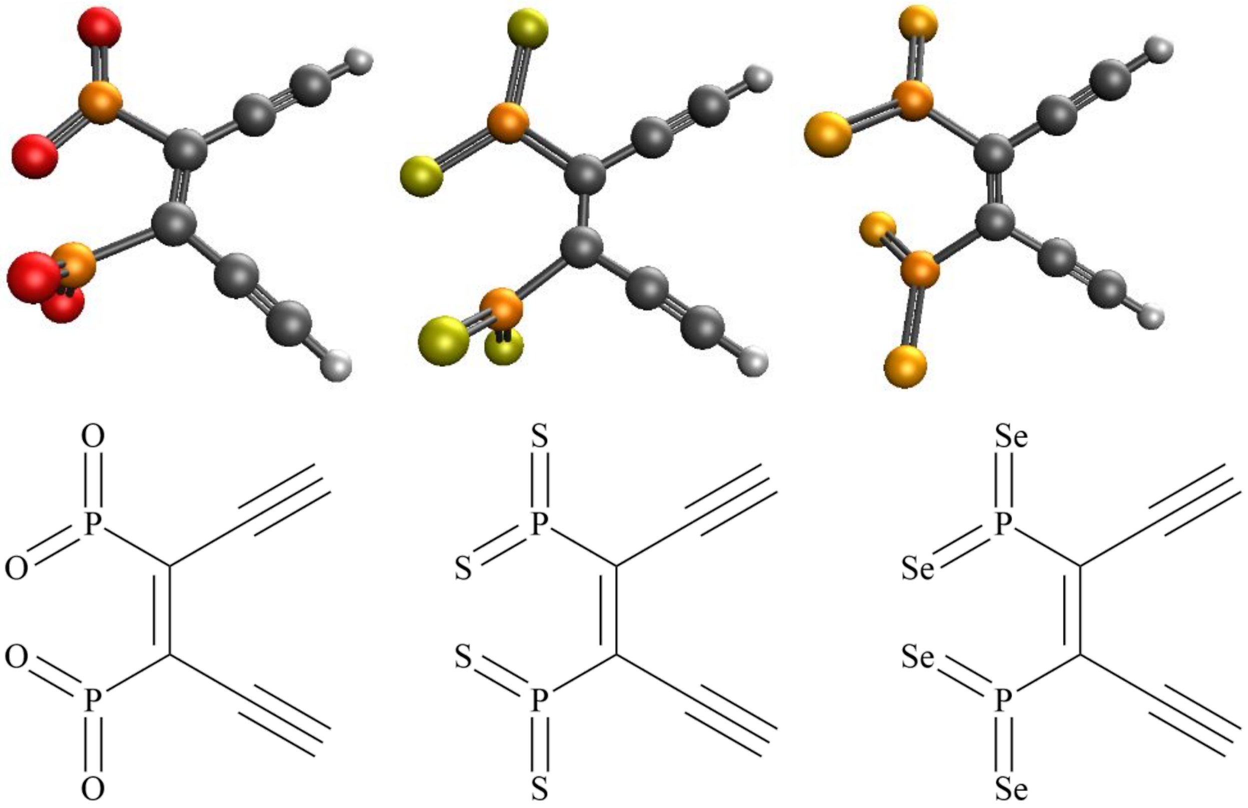

Figure S1

Lowest energy geometry optimized structures of (Z)-bis(**oxide/sulfide/selenide**)-3λ^4,4λ^4-diphosphane-hex-3-ene-1,5-diyne, referenced in the main paper as **R**. This structure is the starting reactant for both reaction pathways. The symmetry of these congeners is C_1. Geometry optimization was performed using the CCSD method with the cc-pVDZ basis set.

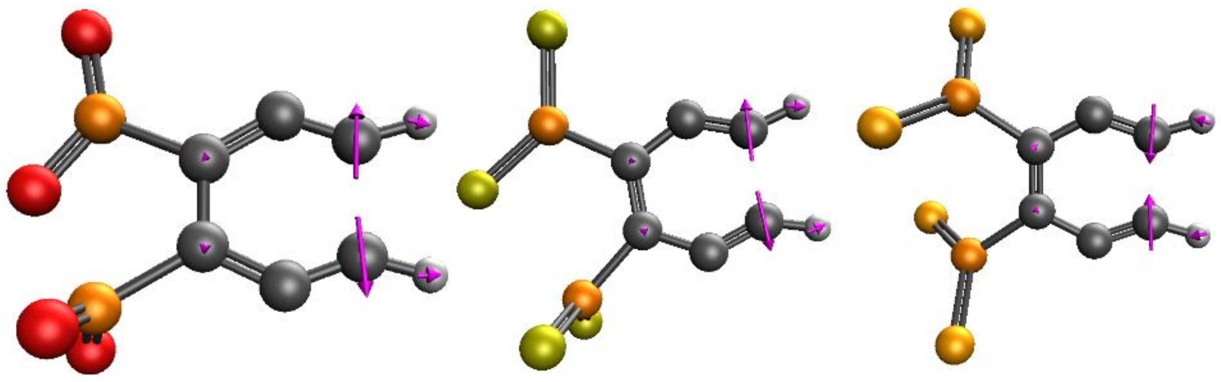

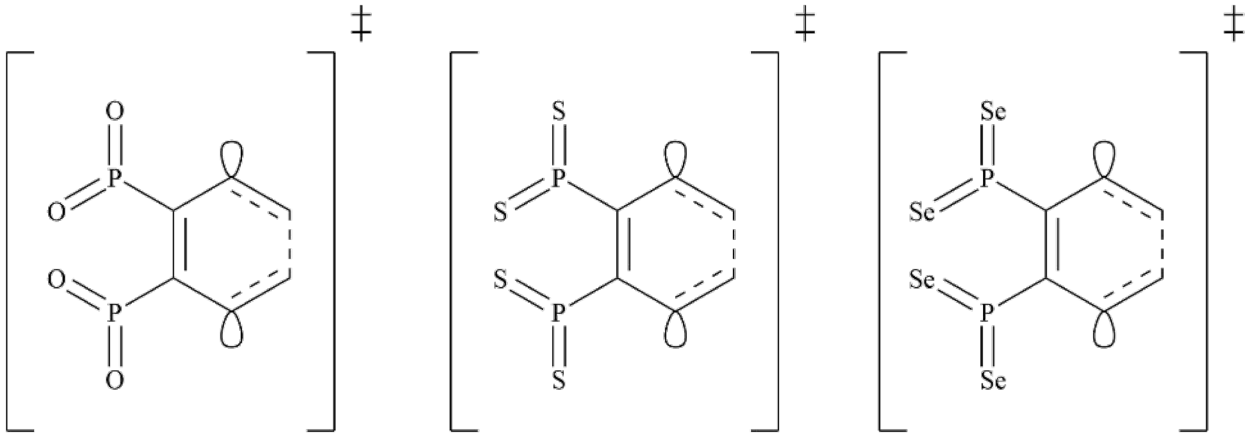

Figure S2

Lowest energy transition state structures of (Z)-bis(**oxide/sulfide/selenide**)-3λ[4],4λ[4]-diphosphane-hex-3-ene-1,5-diyne, referenced in the main paper as $\mathbf{TS_{Berg}}$. This structure is the transition state of the Bergman cyclization from starting structure **R**. The symmetry of these congeners is C_1. Transition state optimization was performed using the CCSD method with the cc-pVDZ basis set.

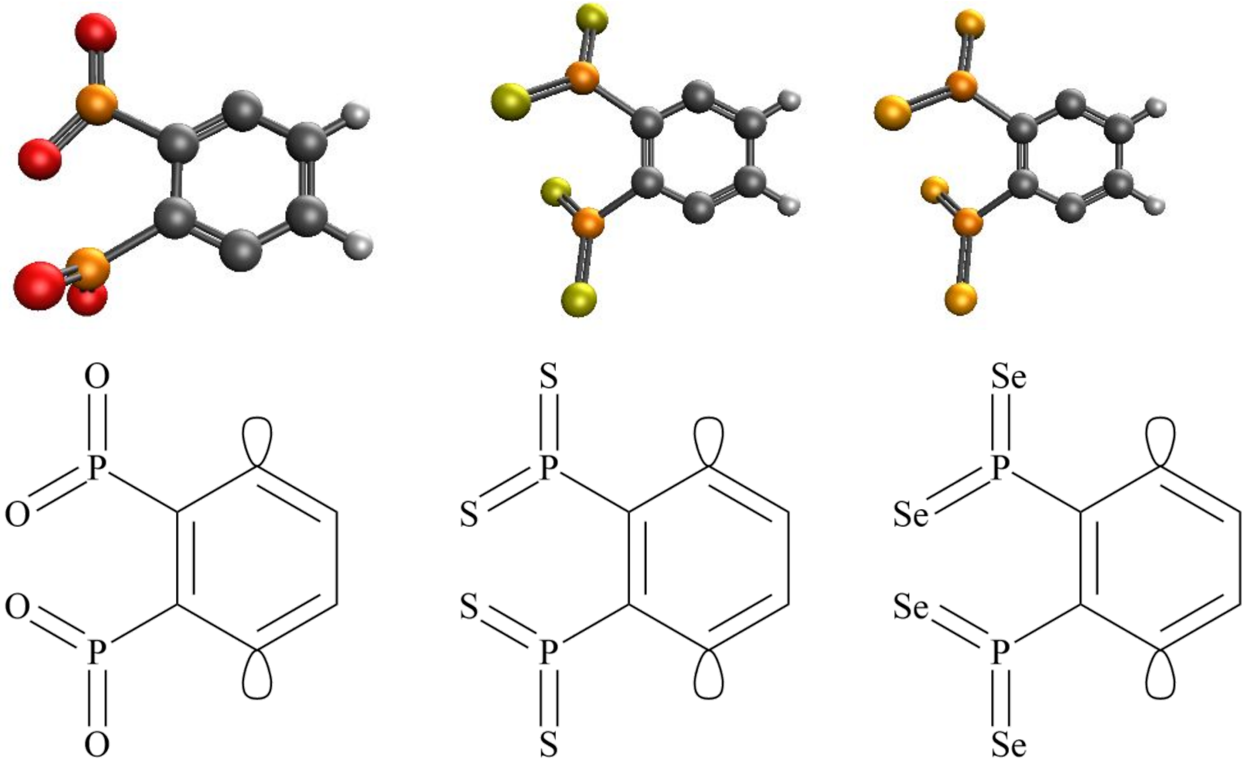

Figure S3

Lowest energy geometry optimized structures of bis(**oxide/sulfide/selenide**)-3λ[4],4λ[4]-diphosphane-2,5-phenyl, referenced in the main paper as $\mathbf{P_{Berg}}$. This structure is the intermediate of the first reaction pathway, in which the Bergman cyclization was the first cyclization performed on structure **R**. The symmetry of the oxygen congener is C_1 while the symmetry of the sulfur and selenium congeners is C_2. Geometry optimization was performed using the CCSD method with the cc-pVDZ basis set.

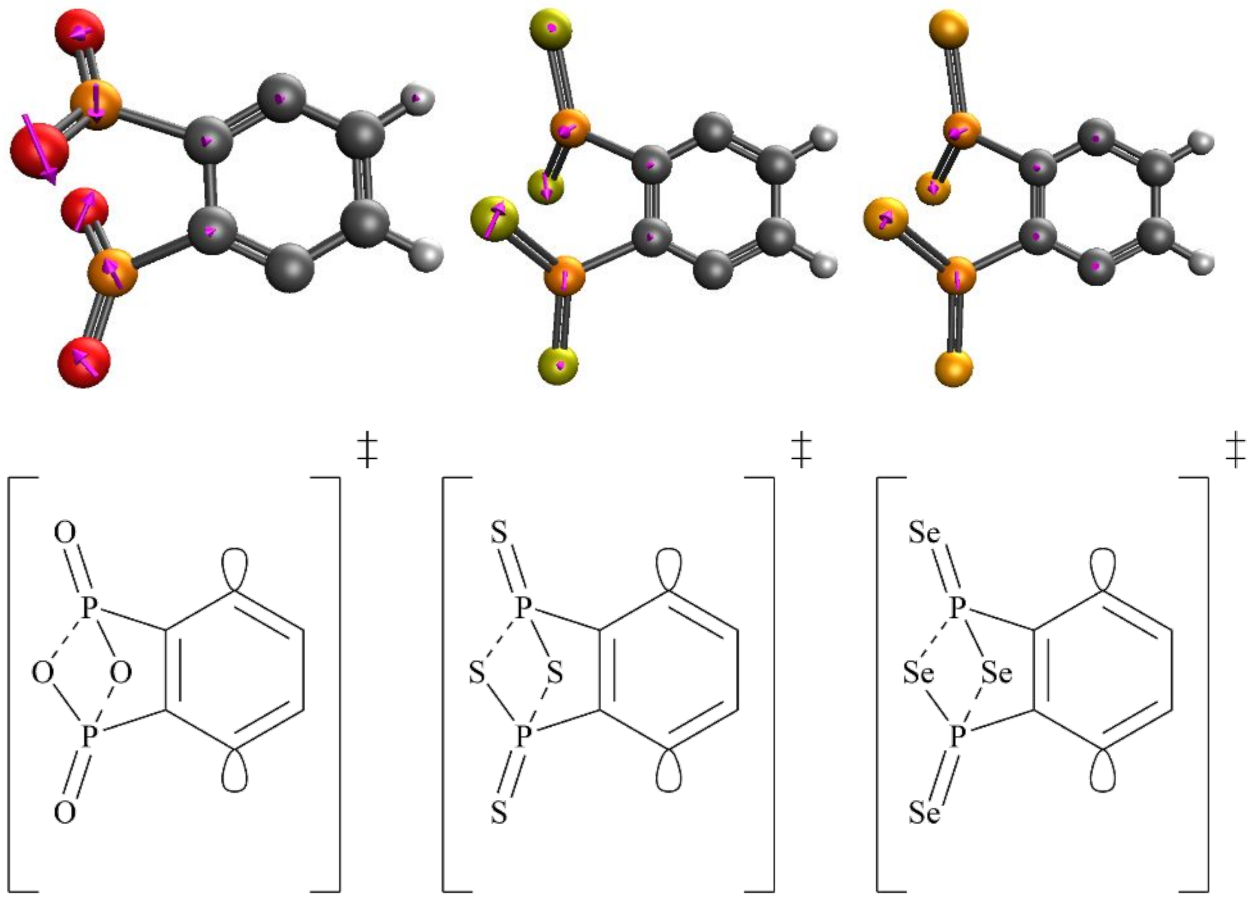

Figure S4

Lowest energy transition state structures of bis(**oxide/sulfide/selenide**)-3λ^4,4λ^4-diphosphane-2,5-phenyl, referenced in the main paper as $\mathbf{TS_{Berg\text{-}chalc}}$. This structure is the transition state of the chalcogen cyclization from structure $\mathbf{P_{Berg}}$. The symmetry of these congeners is C_1. Transition state optimization was performed using the CCSD method with the cc-pVDZ basis set.

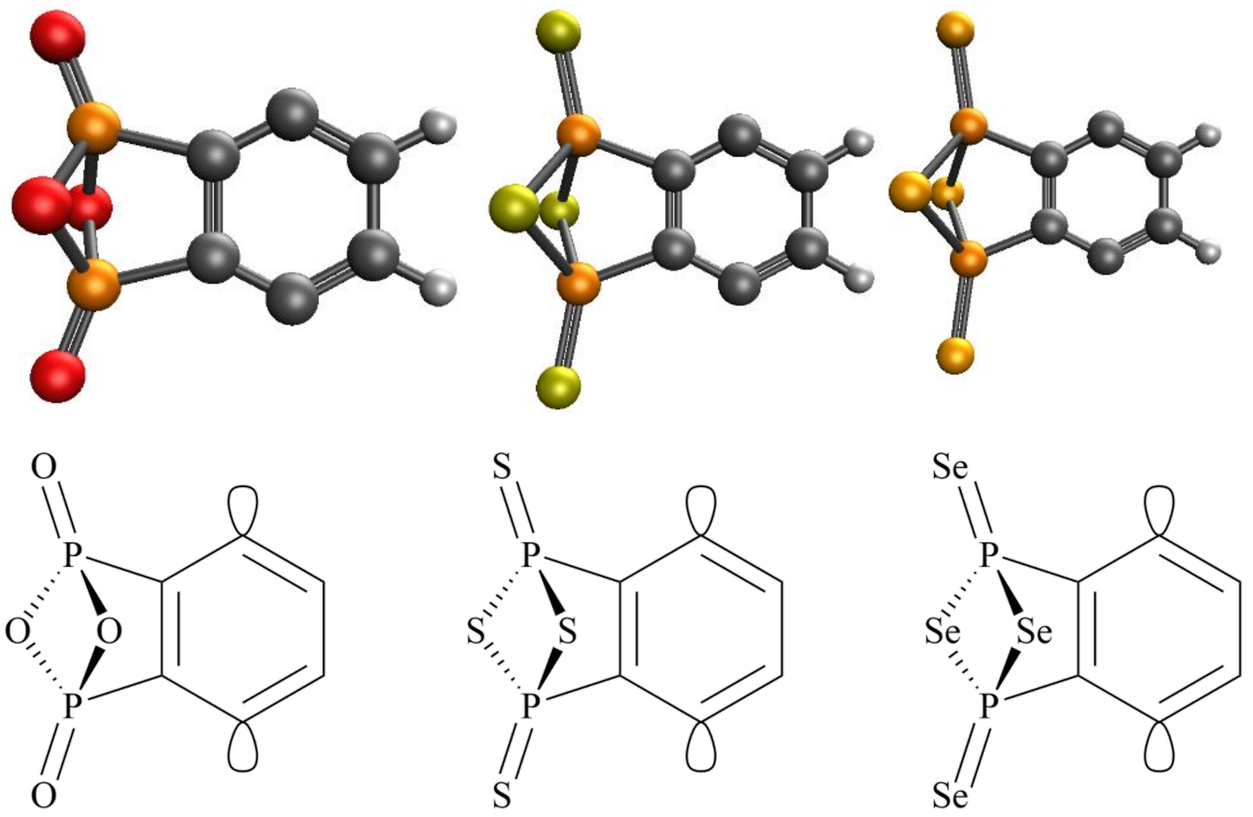

Figure S5

Lowest energy geometry optimized structures di**oxo/thia/selena**diphosphetane-(2,4-di**one/thione/selone**)-bicyclo[2.1.1]-2,5-phenyl, referenced in the main paper as $\mathbf{P_{Berg\text{-}chalc}}$. This structure is the final product of both reaction pathways and contains both the diradical ring as well as the chalcogen-phosphorus ring. The symmetry of these congeners is C_{2v}. Geometry optimization was performed using the CCSD method with the cc-pVDZ basis set.

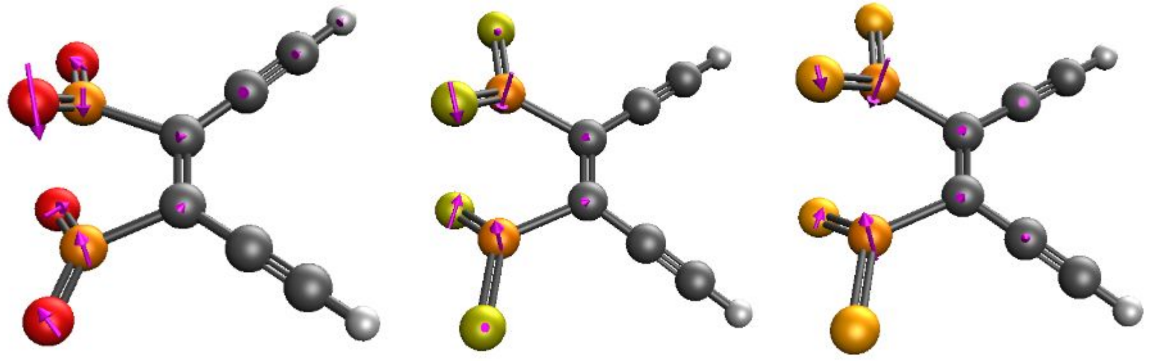

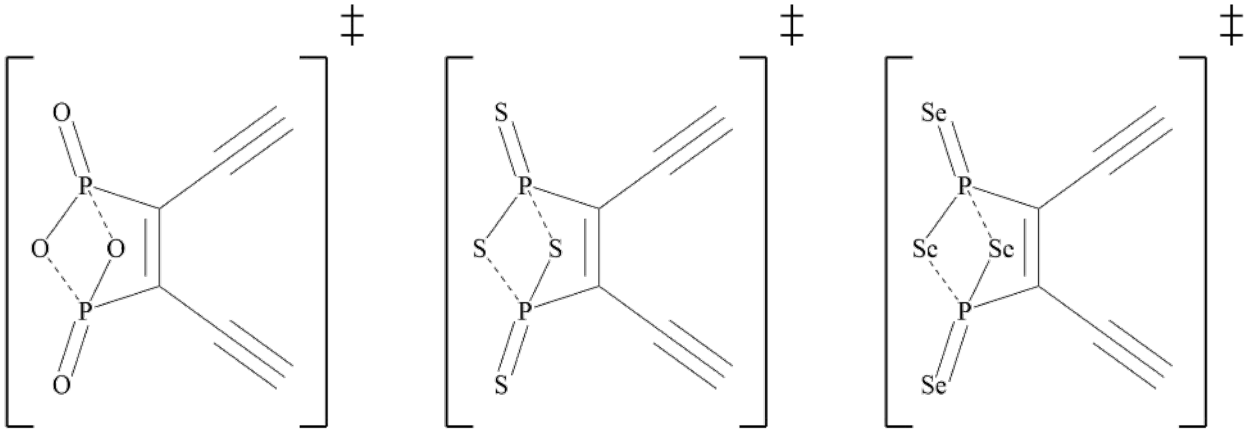

Figure S6

Lowest energy transition state structures of (Z)-bis(**oxide/sulfide/selenide**)-3λ^4,4λ^4-diphosphane-hex-3-ene-1,5-diyne, referenced in the main paper as $\mathbf{TS_{chalc}}$. This structure is the transition state of the chalcogen cyclization from starting structure **R**. The symmetry of these congeners is C_1. Transition state optimization was performed using the CCSD method with the cc-pVDZ basis set.

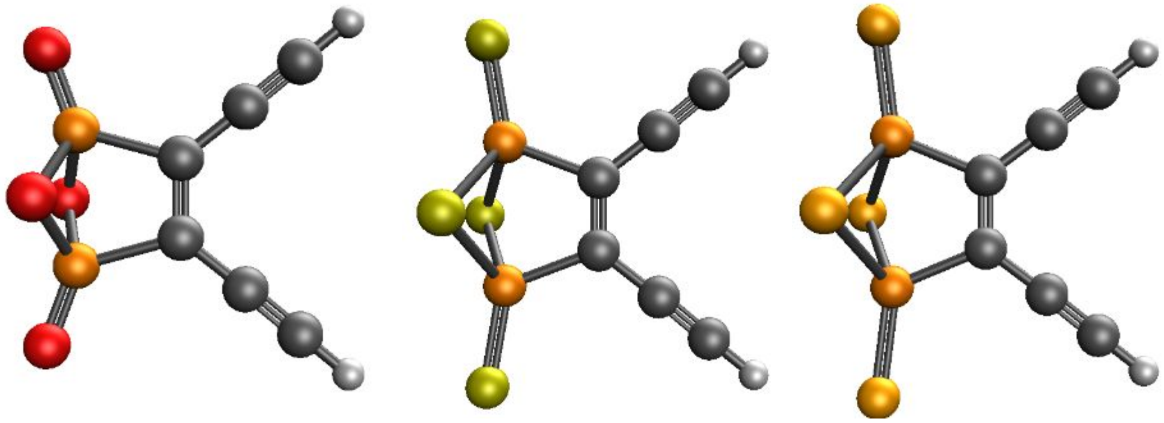

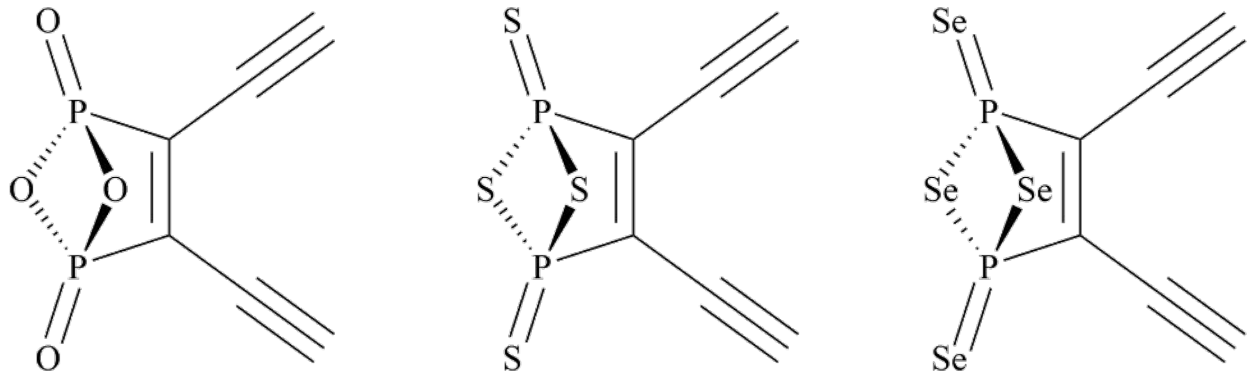

Figure S7

Lowest energy geometry optimized structures di**oxo/thia/selena**diphosphetane-(2,4-di**one/thione/selone**)-bicyclo[2.1.1]-hex-3-ene-1,5-diyne, referenced in the main paper as $\mathbf{P_{chalc}}$. This structure is the intermediate of the second reaction pathway, in which the chalcogen cyclization was the first cyclization performed on structure **R**. The symmetry of these congeners is C_{2v}. Geometry optimization was performed using the CCSD method with the cc-pVDZ basis set.

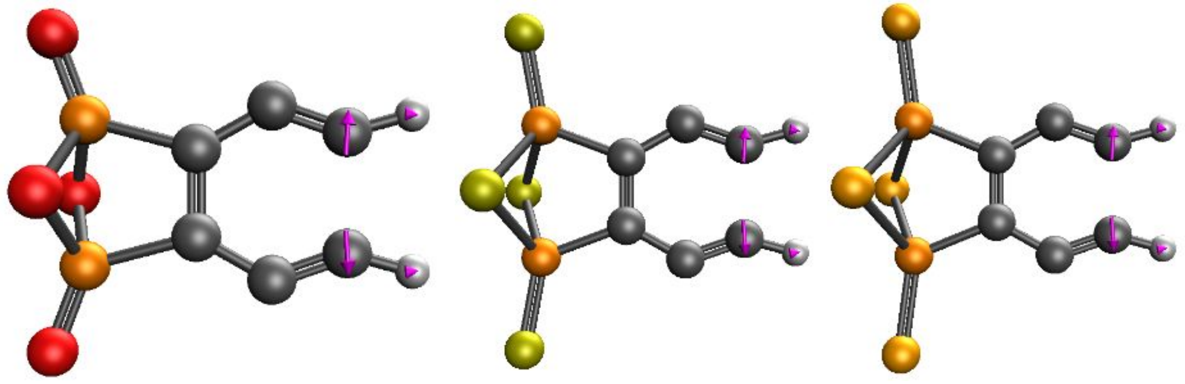

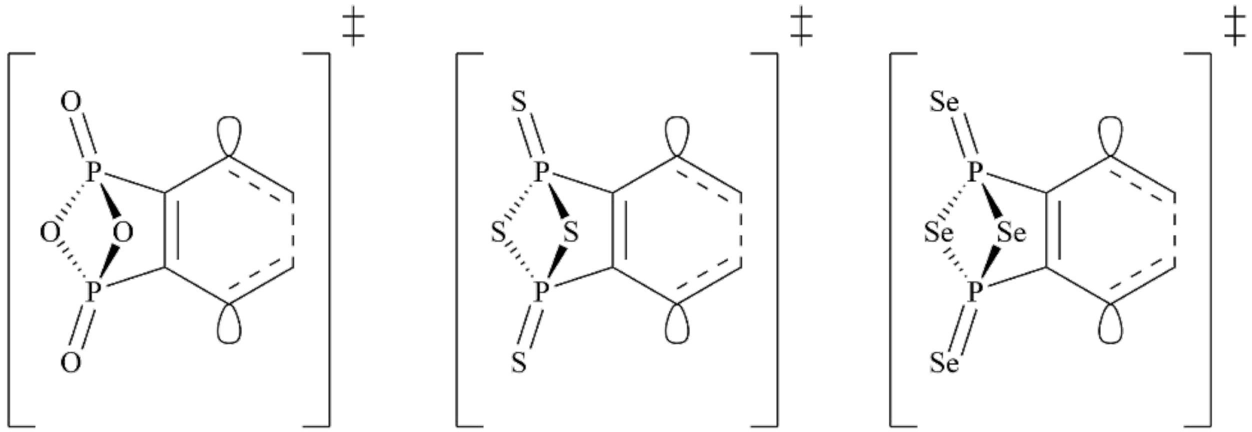

Figure S8

Lowest energy transition state structures of di**oxo/thia/selena**diphosphetane-(2,4-di**one/thione/selone**)-bicyclo[2.1.1]-hex-3-ene-1,5-diyne, referenced in the main paper as $\mathbf{TS_{chalc\text{-}Berg}}$. This structure is the transition state of the Bergman cyclization from structure $\mathbf{P_{chalc}}$. The symmetry of these congeners is C_{2v}. Transition state optimization was performed using the CCSD method with the cc-pVDZ basis set.

Table S1. Bond lengths (Å) and angles (°) for the congeners of the optimized structure **R**, optimized at the CCSD/cc-pVDZ level of theory.

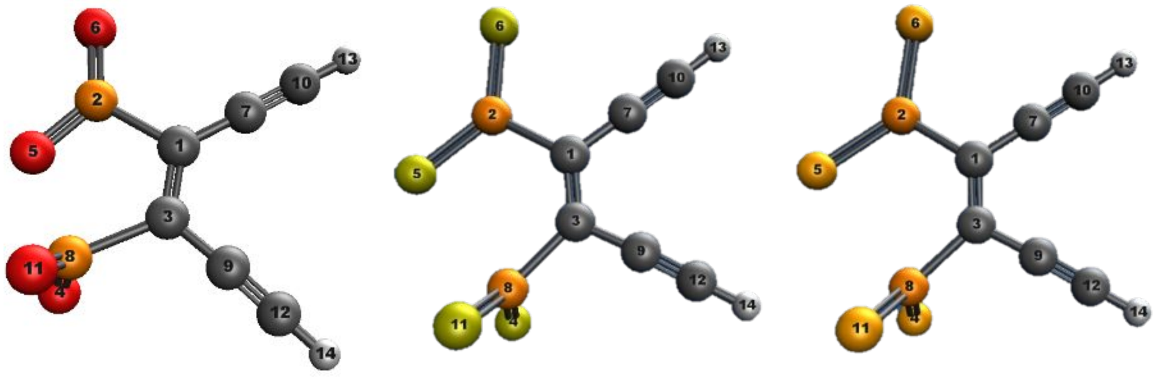

Parameter[a]	Oxygen	Sulfur	Selenium
$R_{2\text{-}6}$ (Å)	1.48	1.92	2.07
$R_{2\text{-}5}$ (Å)	1.52	1.94	2.08
$R_{1\text{-}2}$ (Å)	1.78	1.84	1.84
$R_{1\text{-}3}$ (Å)	1.37	1.37	1.37
$R_{1\text{-}7}$ (Å)	1.44	1.44	1.44
$R_{7\text{-}10}$ (Å)	1.22	1.22	1.22
$R_{10\text{-}13}$ (Å)	1.08	1.08	1.08
$\angle_{6\text{-}2\text{-}1}$ (°)	122.7	115.1	115.3
$\angle_{11\text{-}8\text{-}3}$ (°)	110.1	111.8	111.6
$\angle_{6\text{-}2\text{-}5}$ (°)	130.4	131.4	130.8
$\angle_{4\text{-}8\text{-}11}$ (°)	134.8	133.9	133.5
$\angle_{5\text{-}2\text{-}1}$ (°)	106.9	113.5	113.9
$\angle_{4\text{-}8\text{-}3}$ (°)	110.1	111.8	111.5
$\angle_{2\text{-}1\text{-}3}$ (°)	111.9	123.2	124.2
$\angle_{8\text{-}3\text{-}1}$ (°)	121.3	129.5	130.3
$\angle_{2\text{-}1\text{-}7}$ (°)	121.0	116.0	115.6
$\angle_{8\text{-}3\text{-}9}$ (°)	116.6	110.0	109.7
$\angle_{3\text{-}1\text{-}7}$ (°)	127.1	120.8	120.3
$\angle_{1\text{-}3\text{-}9}$ (°)	122.1	120.5	120.0
$\angle_{1\text{-}7\text{-}10}$ (°)	179.5	179.9	179.9
$\angle_{3\text{-}9\text{-}12}$ (°)	177.3	176.9	177.3
$\angle_{7\text{-}10\text{-}13}$ (°)	179.9	179.7	179.7
$\angle_{9\text{-}12\text{-}14}$ (°)	179.4	179.2	179.3

(a) Only unique bond lengths are shown

Table S2. Bond lengths (Å) and angles (°) for the congeners of the optimized transition state $\mathbf{TS_{Berg}}$, optimized at the UCCSD/cc-pVDZ level of theory.

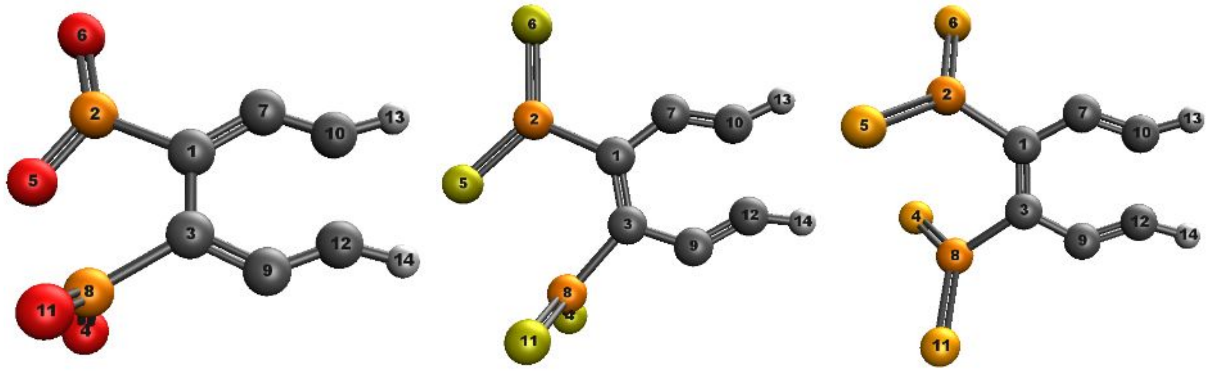

Parameter(a-b)	Oxygen	Sulfur	Selenium
R_{2-6} (Å)	1.48	1.93	2.07
R_{2-5} (Å)	1.52	1.94	2.08
R_{1-2} (Å)	1.77	1.83	1.85
R_{1-3} (Å)	1.42	1.42	1.41
R_{1-7} (Å)	1.41	1.42	1.41
R_{7-10} (Å)	1.28	1.28	1.28
R_{10-13} (Å)	1.09	1.09	1.09
R_{10-12} (Å)	1.92	1.90	1.91
$\angle_{6-2-1}$ (°)	122.7	114.5	113.3
$\angle_{11-8-3}$ (°)	110.2	112.1	113.3
$\angle_{6-2-5}$ (°)	130.5	131.4	133.5
$\angle_{4-8-11}$ (°)	134.7	133.8	133.5
$\angle_{5-2-1}$ (°)	106.8	114.1	113.0
$\angle_{4-8-3}$ (°)	110.2	112.1	113.0
$\angle_{2-1-3}$ (°)	112.0	123.6	124.1
$\angle_{8-3-1}$ (°)	120.5	128.9	124.0
$\angle_{2-1-7}$ (°)	127.0	118.7	117.8
$\angle_{8-3-9}$ (°)	123.1	113.1	117.9
$\angle_{3-1-7}$ (°)	121.0	117.7	118.1
$\angle_{1-3-9}$ (°)	116.5	118.0	118.1
$\angle_{1-7-10}$ (°)	130.0	133.3	132.9
$\angle_{3-9-12}$ (°)	133.4	132.9	132.9
$\angle_{7-10-13}$ (°)	145.0	144.9	144.8
$\angle_{9-12-14}$ (°)	144.3	144.4	144.8

(a) Only unique bond lengths are shown

Table S3. Bond lengths (Å) and angles (°) for the congeners of the optimized structure $\mathbf{P_{Berg}}$, optimized at the UCCSD/cc-pVDZ level of theory.

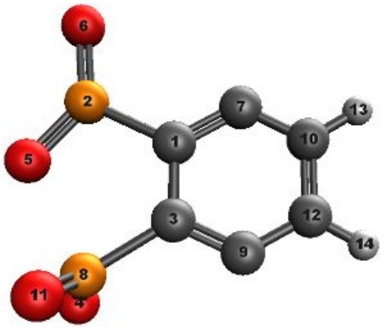

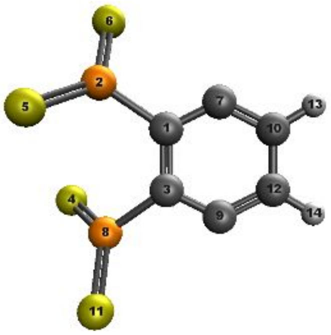

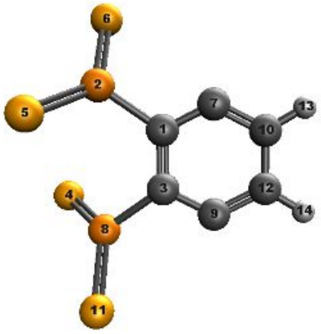

Parameter[(a)]	Oxygen	Sulfur	Selenium
$\boldsymbol{R}_{2\text{-}6}$ (Å)	1.48	1.92	2.07
$\boldsymbol{R}_{2\text{-}5}$ (Å)	1.50	1.93	2.07
$\boldsymbol{R}_{1\text{-}2}$ (Å)	1.79	1.84	1.84
$\boldsymbol{R}_{1\text{-}3}$ (Å)	1.43	1.43	1.43
$\boldsymbol{R}_{1\text{-}7}$ (Å)	1.39	1.38	1.39
$\boldsymbol{R}_{7\text{-}10}$ (Å)	1.39	1.39	1.39
$\boldsymbol{R}_{10\text{-}13}$ (Å)	1.10	1.10	1.10
$\boldsymbol{R}_{10\text{-}12}$ (Å)	1.42	1.41	1.41
$\angle_{6\text{-}2\text{-}1}$ (°)	120.0	113.6	113.5
$\angle_{11\text{-}8\text{-}3}$ (°)	111.6	113.6	113.5
$\angle_{6\text{-}2\text{-}5}$ (°)	132.7	133.3	133.7
$\angle_{4\text{-}8\text{-}11}$ (°)	134.8	133.3	133.7
$\angle_{5\text{-}2\text{-}1}$ (°)	107.3	112.9	112.5
$\angle_{4\text{-}8\text{-}3}$ (°)	111.6	112.9	112.5
$\angle_{2\text{-}1\text{-}3}$ (°)	115.4	124.6	124.9
$\angle_{8\text{-}3\text{-}1}$ (°)	121.8	124.6	124.9
$\angle_{2\text{-}1\text{-}7}$ (°)	125.5	118.2	118.0
$\angle_{8\text{-}3\text{-}9}$ (°)	122.5	118.2	118.0
$\angle_{3\text{-}1\text{-}7}$ (°)	119.1	116.8	116.8
$\angle_{1\text{-}3\text{-}9}$ (°)	115.7	116.8	116.8
$\angle_{1\text{-}7\text{-}10}$ (°)	124.5	126.2	126.2
$\angle_{3\text{-}9\text{-}12}$ (°)	125.7	126.2	126.2
$\angle_{7\text{-}10\text{-}13}$ (°)	122.6	122.2	122.2
$\angle_{9\text{-}12\text{-}14}$ (°)	121.9	122.2	122.2
$\angle_{13\text{-}10\text{-}12}$ (°)	120.4	120.8	120.8
$\angle_{14\text{-}12\text{-}10}$ (°)	120.0	120.8	120.8

(a) Only unique bond lengths are shown

Table S4. Bond lengths (Å) and angles (°) for the congeners of the optimized transition state $\mathbf{TS_{Berg\text{-}chalc}}$, optimized at the UCCSD/cc-pVDZ level of theory.

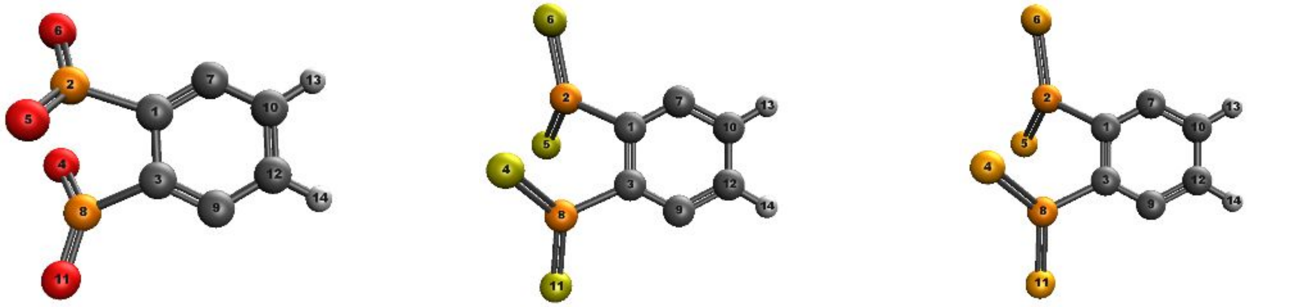

Parameter[(a)]	Oxygen	Sulfur	Selenium
$R_{2\text{-}6}$ (Å)	1.48	1.92	2.07
$R_{2\text{-}5}$ (Å)	1.51	1.99	2.14
$R_{2\text{-}4}$ (Å)	2.15	2.78	2.96
$R_{5\text{-}8}$ (Å)	2.50	2.78	2.96
$R_{1\text{-}2}$ (Å)	1.84	1.84	1.84
$R_{1\text{-}3}$ (Å)	1.44	1.43	1.43
$R_{1\text{-}7}$ (Å)	1.37	1.37	1.38
$R_{7\text{-}10}$ (Å)	1.40	1.40	1.40
$R_{10\text{-}13}$ (Å)	1.10	1.10	1.10
$R_{10\text{-}12}$ (Å)	1.41	1.41	1.41
$\angle_{6\text{-}2\text{-}1}$ (°)	119.1	118.6	117.5
$\angle_{11\text{-}8\text{-}3}$ (°)	122.9	118.6	117.5
$\angle_{6\text{-}2\text{-}5}$ (°)	134.1	131.1	131.2
$\angle_{4\text{-}8\text{-}11}$ (°)	132.0	131.1	131.2
$\angle_{5\text{-}2\text{-}1}$ (°)	103.6	105.1	105.6
$\angle_{4\text{-}8\text{-}3}$ (°)	103.0	105.1	105.6
$\angle_{2\text{-}1\text{-}3}$ (°)	109.8	114.2	116.3
$\angle_{8\text{-}3\text{-}1}$ (°)	109.2	114.2	116.3
$\angle_{2\text{-}1\text{-}7}$ (°)	132.2	127.3	125.4
$\angle_{8\text{-}3\text{-}9}$ (°)	131.2	127.3	125.4
$\angle_{3\text{-}1\text{-}7}$ (°)	117.0	118.0	117.8
$\angle_{1\text{-}3\text{-}9}$ (°)	119.0	118.0	117.8
$\angle_{1\text{-}7\text{-}10}$ (°)	124.3	124.2	124.6
$\angle_{3\text{-}9\text{-}12}$ (°)	123.6	124.2	124.6
$\angle_{7\text{-}10\text{-}13}$ (°)	121.9	122.0	122.0
$\angle_{9\text{-}12\text{-}14}$ (°)	122.1	122.0	122.0
$\angle_{13\text{-}10\text{-}12}$ (°)	119.8	120.2	120.3
$\angle_{14\text{-}12\text{-}10}$ (°)	120.1	120.2	120.3

(a) Only unique bond lengths are shown

Table S5. Bond lengths (Å) and angles (°) for the congeners of the optimized structure $\mathbf{P_{Berg\text{-}chalc}}$, optimized at the UCCSD/cc-pVDZ level of theory.

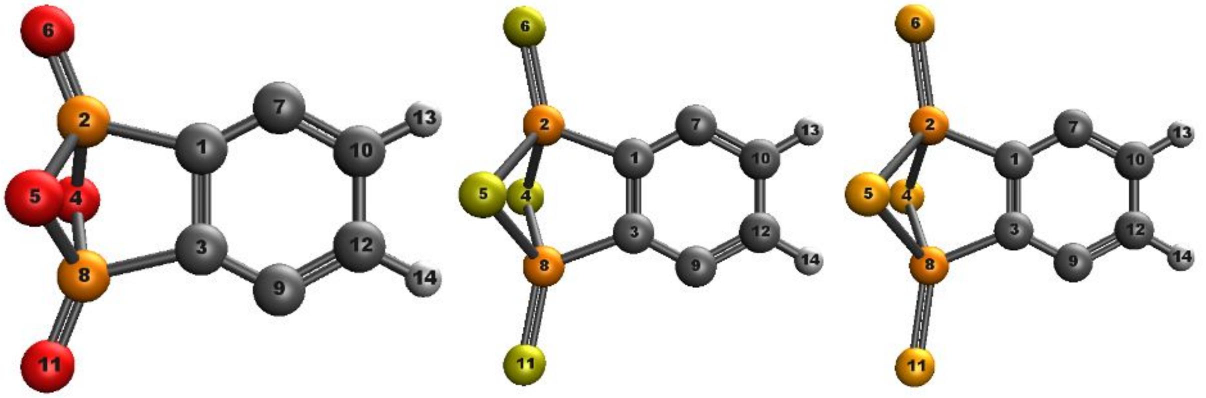

Parameter[(a-b)]	Oxygen	Sulfur	Selenium
$R_{2\text{-}6}$ (Å)	1.47	1.93	2.08
$R_{2\text{-}5}$ (Å)	1.69	2.18	2.33
$R_{1\text{-}2}$ (Å)	1.84	1.86	1.86
$R_{1\text{-}3}$ (Å)	1.53	1.44	1.43
$R_{1\text{-}7}$ (Å)	1.34	1.37	1.37
$R_{7\text{-}10}$ (Å)	1.39	1.40	1.40
$R_{10\text{-}13}$ (Å)	1.09	1.10	1.10
$R_{10\text{-}12}$ (Å)	1.44	1.41	1.41
$\angle_{6\text{-}2\text{-}1}$ (°)	124.8	121.4	120.3
$\angle_{6\text{-}2\text{-}5}$ (°)	123.7	123.3	123.8
$\angle_{2\text{-}4\text{-}8}$ (°)	88.1	77.3	75.2
$\angle_{4\text{-}2\text{-}5}$ (°)	85.0	90.0	90.7
$\angle_{5\text{-}2\text{-}1}$ (°)	94.7	95.4	95.2
$\angle_{2\text{-}1\text{-}3}$ (°)	103.0	110.4	112.4
$\angle_{2\text{-}1\text{-}7}$ (°)	139.4	131.4	129.6
$\angle_{3\text{-}1\text{-}7}$ (°)	117.6	118.2	118.1
$\angle_{1\text{-}7\text{-}10}$ (°)	123.9	123.8	124.0
$\angle_{7\text{-}10\text{-}13}$ (°)	124.5	121.9	121.9
$\angle_{7\text{-}10\text{-}12}$ (°)	118.5	118.1	117.9
$\angle_{13\text{-}10\text{-}12}$ (°)	117.0	120.0	120.1

(a) Only unique bond lengths are shown
(b) Only unique angles are shown

Table S6. Bond lengths (Å) and angles (°) for the congeners of the optimized transition state $\mathbf{TS_{chalc}}$, optimized at the UCCSD/cc-pVDZ level of theory.

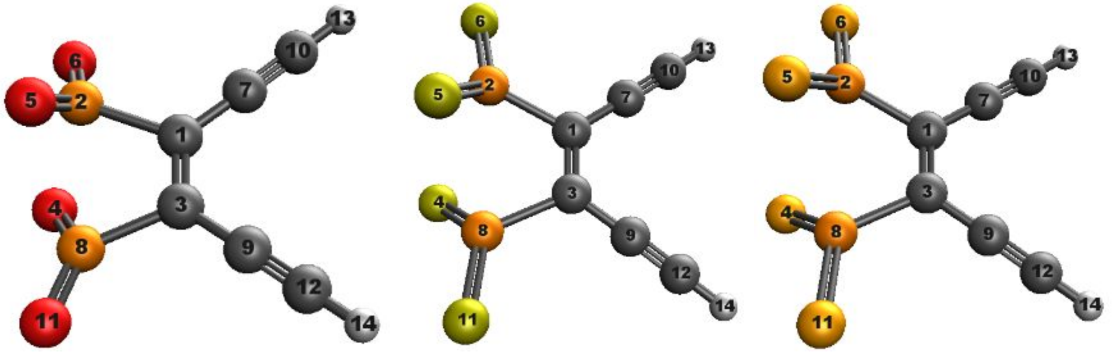

Parameter[a]	Oxygen	Sulfur	Selenium
$R_{2\text{-}6}$ (Å)	1.48	1.92	2.07
$R_{2\text{-}5}$ (Å)	1.51	1.99	2.13
$R_{2\text{-}4}$ (Å)	2.01	2.79	2.97
$R_{5\text{-}8}$ (Å)	2.58	2.79	2.97
$R_{1\text{-}2}$ (Å)	1.87	1.86	1.87
$R_{1\text{-}3}$ (Å)	1.37	1.36	1.36
$R_{1\text{-}7}$ (Å)	1.43	1.43	1.43
$R_{7\text{-}10}$ (Å)	1.23	1.23	1.23
$R_{10\text{-}13}$ (Å)	1.08	1.08	1.08
$\angle_{6\text{-}2\text{-}1}$ (°)	117.2	117.8	117.1
$\angle_{11\text{-}8\text{-}3}$ (°)	123.9	117.8	117.1
$\angle_{6\text{-}2\text{-}5}$ (°)	134.4	131.3	131.1
$\angle_{4\text{-}8\text{-}11}$ (°)	130.4	131.3	131.1
$\angle_{5\text{-}2\text{-}1}$ (°)	102.8	105.7	106.1
$\angle_{4\text{-}8\text{-}3}$ (°)	104.5	105.7	106.0
$\angle_{2\text{-}1\text{-}3}$ (°)	111.2	114.	116.4
$\angle_{8\text{-}3\text{-}1}$ (°)	108.3	114.6	116.5
$\angle_{2\text{-}1\text{-}7}$ (°)	122.7	119.2	118.5
$\angle_{8\text{-}3\text{-}9}$ (°)	122.5	119.2	118.4
$\angle_{3\text{-}1\text{-}7}$ (°)	125.3	125.9	124.8
$\angle_{1\text{-}3\text{-}9}$ (°)	129.1	125.9	124.8
$\angle_{1\text{-}7\text{-}10}$ (°)	178.1	178.5	178.7
$\angle_{3\text{-}9\text{-}12}$ (°)	179.1	178.6	178.7
$\angle_{7\text{-}10\text{-}13}$ (°)	179.7	179.6	179.6
$\angle_{9\text{-}12\text{-}14}$ (°)	179.7	179.6	179.6

(a) Only unique bond lengths are shown

Table S7. Bond lengths (Å) and angles (°) for the congeners of the optimized structure $\mathbf{P_{chalc}}$, optimized at the UCCSD/cc-pVDZ level of theory.

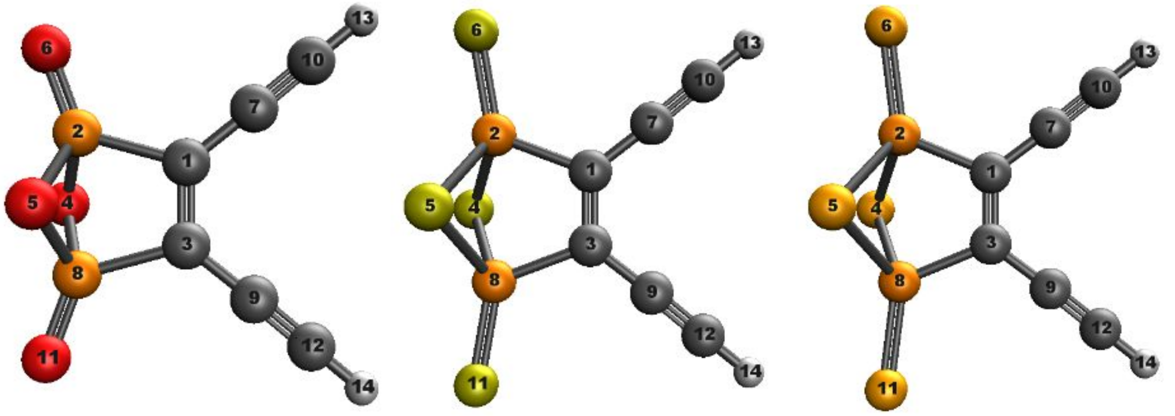

Parameter[(a-b)]	Oxygen	Sulfur	Selenium
$\boldsymbol{R}_{2\text{-}6}$ (Å)	1.47	1.93	2.08
$\boldsymbol{R}_{2\text{-}5}$ (Å)	1.69	2.18	2.33
$\boldsymbol{R}_{1\text{-}2}$ (Å)	1.86	1.89	1.89
$\boldsymbol{R}_{1\text{-}3}$ (Å)	1.37	1.36	1.36
$\boldsymbol{R}_{1\text{-}7}$ (Å)	1.43	1.43	1.43
$\boldsymbol{R}_{7\text{-}10}$ (Å)	1.23	1.23	1.23
$\boldsymbol{R}_{10\text{-}13}$ (Å)	1.08	1.08	1.08
$\angle_{6\text{-}2\text{-}1}$ (°)	125.3	120.5	119.7
$\angle_{6\text{-}2\text{-}5}$ (°)	123.9	123.4	123.7
$\angle_{2\text{-}4\text{-}8}$ (°)	87.3	76.3	74.2
$\angle_{4\text{-}2\text{-}5}$ (°)	84.9	90.3	91.0
$\angle_{5\text{-}2\text{-}1}$ (°)	94.1	95.7	95.6
$\angle_{2\text{-}1\text{-}3}$ (°)	105.0	110.7	112.6
$\angle_{2\text{-}1\text{-}7}$ (°)	126.5	121.4	120.5
$\angle_{3\text{-}1\text{-}7}$ (°)	128.5	127.9	126.9
$\angle_{1\text{-}7\text{-}10}$ (°)	178.8	178.2	178.2
$\angle_{7\text{-}10\text{-}13}$ (°)	179.8	179.4	179.4

(a) Only unique bond lengths are shown
(b) Only unique angles are shown

Table S8. Bond lengths (Å) and angles (°) for the congeners of the optimized transition state $\mathbf{TS}_{\mathbf{chalc\text{-}Berg}}$, optimized at the UCCSD/cc-pVDZ level of theory.

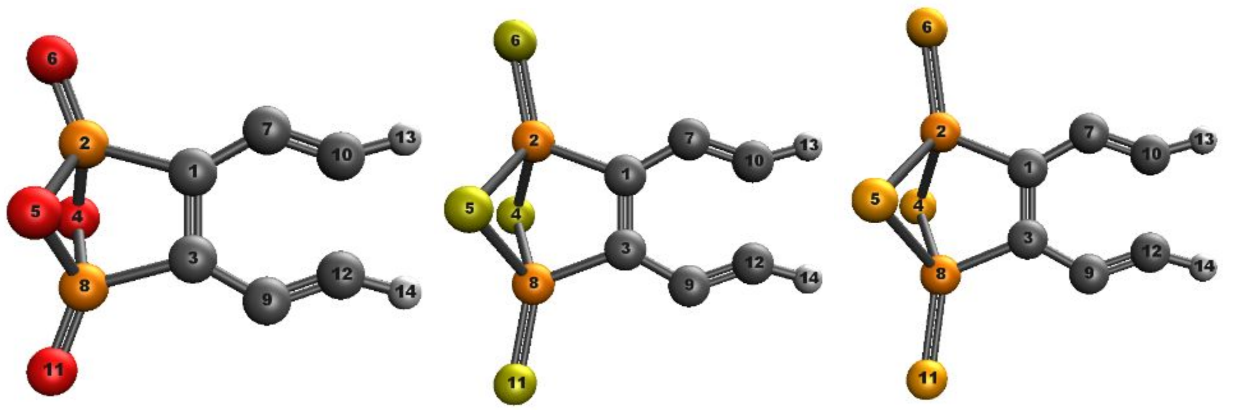

Parameter[(a-b)]	Oxygen	Sulfur	Selenium
$\boldsymbol{R}_{2-6}$ **(Å)**	1.47	1.93	2.08
$\boldsymbol{R}_{2-5}$ **(Å)**	1.69	2.18	2.33
$\boldsymbol{R}_{1-2}$ **(Å)**	1.85	1.87	1.87
$\boldsymbol{R}_{1-3}$ **(Å)**	1.42	1.41	1.41
$\boldsymbol{R}_{1-7}$ **(Å)**	1.40	1.40	1.40
$\boldsymbol{R}_{7-10}$ **(Å)**	1.28	129	1.28
$\boldsymbol{R}_{10-13}$ **(Å)**	1.09	1.09	1.09
$\boldsymbol{R}_{10-12}$ **(Å)**	1.95	1.94	1.93
$\angle_{6-2-1}$ **(°)**	125.3	120.8	119.8
$\angle_{6-2-5}$ **(°)**	123.8	123.3	123.7
$\angle_{2-4-8}$ **(°)**	87.7	76.7	74.6
$\angle_{4-2-5}$ **(°)**	84.9	90.1	90.8
$\angle_{5-2-1}$ **(°)**	94.3	95.8	95.7
$\angle_{2-1-3}$ **(°)**	104.5	110.4	112.3
$\angle_{2-1-7}$ **(°)**	136.1	130.1	128.3
$\angle_{3-1-7}$ **(°)**	119.4	119.5	119.4
$\angle_{1-7-10}$ **(°)**	131.4	131.4	131.3
$\angle_{7-10-13}$ **(°)**	145.5	145.4	145.2
$\angle_{7-10-12}$ **(°)**	109.2	109.2	109.3
$\angle_{13-10-12}$ **(°)**	105.3	105.5	105.6

(a) Only unique bond lengths are shown
(b) Only unique angles are shown

a.

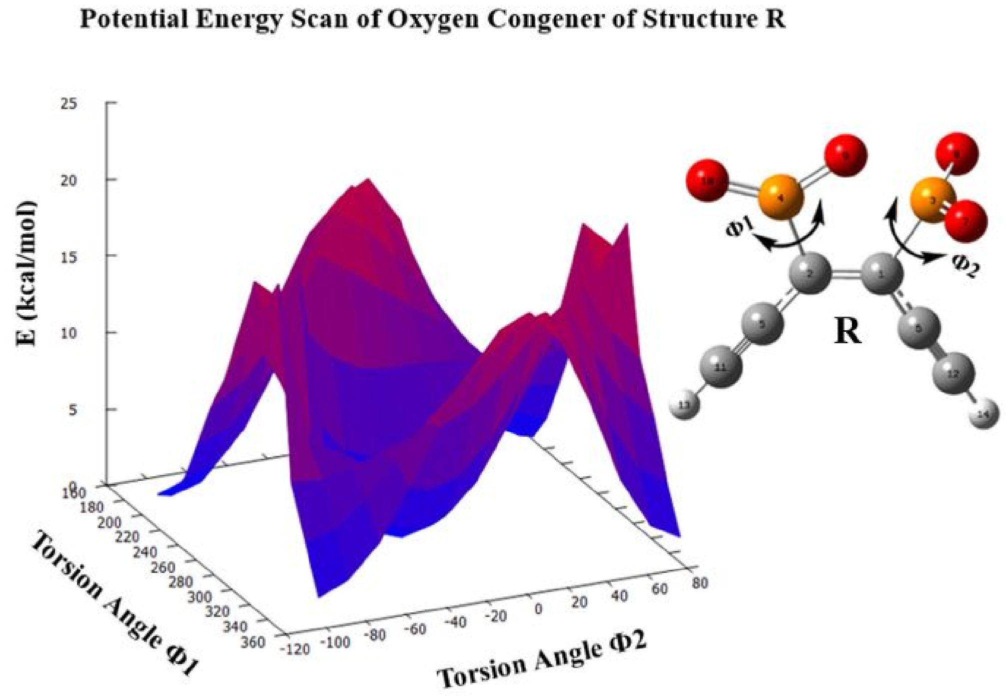
Potential Energy Scan of Oxygen Congener of Structure R
E (kcal/mol)
Torsion Angle Φ1
Torsion Angle Φ2
Φ1
Φ2
R

b.

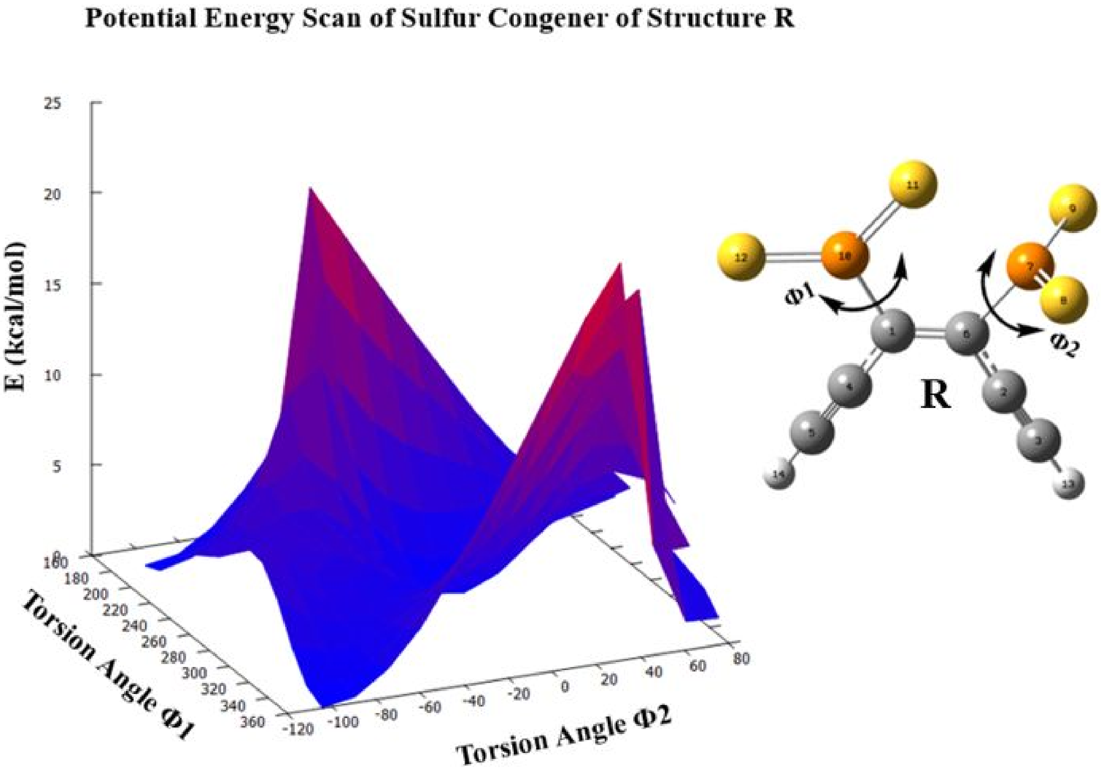
Potential Energy Scan of Sulfur Congener of Structure R
E (kcal/mol)
Torsion Angle Φ1
Torsion Angle Φ2
Φ1
Φ2
R

c.

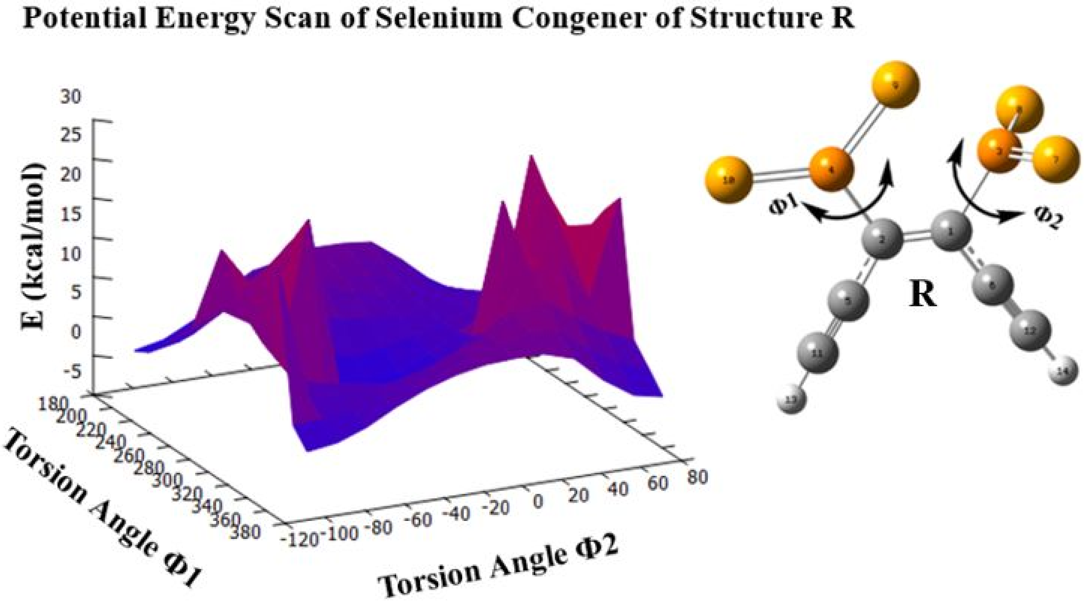

Figure S9. Potential energy scan of structure **R** at the UHF/6-31G* level of theory. **(a)** For the oxygen congener, the lowest energy structure occurs at 179.993 degrees (Φ1) and -103.481 degrees (Φ2) **(b)** For the sulfur congener, the lowest energy structure occurs at 179.880 degrees (Φ1) and -104.443 degrees (Φ2 **(c)** For the selenium congener, lowest energy structure occurs at 181.786 degrees (Φ1) and -100.334 degrees (Φ2).

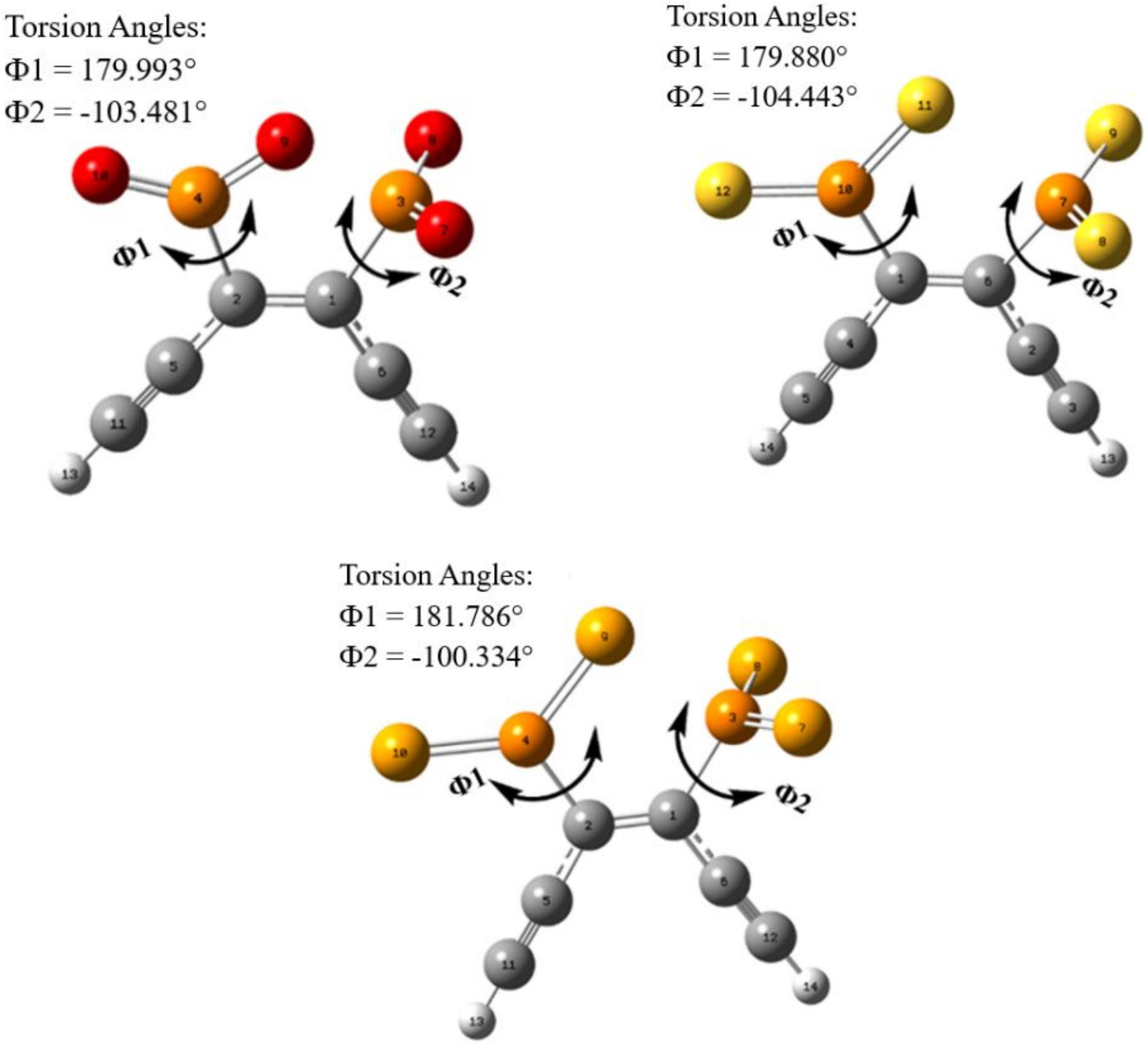

Figure S10. Lowest energy congeners of structure **R**, optimized at the UHF/6-31G* level of theory.

Table S9. Absolute energies (a.u.) and ⟨S^2⟩ values for the singlet reactants, transition states and diradical products for the oxygen, sulfur and selenium chalcogens at the BS-(U)CCSD/DZ level of theory.

	E (a.u.)	S^2
Oxygen		
R	-1210.91173058903	0*
TS_Berg	-1210.85651409663	0.00
P_Berg	-1210.90206410811	1.80
TS_Berg-chalc	-1210.89124825451	1.84
P_Berg-chalc	-1210.91062140493	0.00
TS_chalc	-1210.89942006658	0.00
P_chalc	-1210.93937540930	0*
TS_chalc-Berg	-1210.88345139243	0.00
Sulfur		
R	-2501.42113506204	0*
TS_Berg	-2501.36562818144	0.00
P_Berg	-2501.41523018523	1.85
TS_Berg-chalc	-2501.40022196345	1.85
P_Berg-chalc	-2501.42989852328	1.85
TS_chalc	-2501.40732804410	0.00
P_chalc	-2501.43793312160	0*
TS_chalc-Berg	-2501.38056719193	0.00
Selenium		
R	-10510.5353998234	0*
TS_Berg	-10510.4812504299	0.00
P_Berg	-10510.5298703978	2.32
TS_Berg-chalc	-10510.5187420722	1.87
P_Berg-chalc	-10510.5498177967	1.85
TS_chalc	-10510.5250841610	0.00
P_chalc	-10510.5571711828	0*
TS_chalc-Berg	-10510.4998479420	0.00

*Computed using RHF which is an spin eigenfunction so results are spin pure.

Table S10. Activation energy (E_a) and energy of reaction (E_{rxn}) values (kcal/mol) for the Bergman cyclization and the formation of the chalcogen-phosphorus rings relative to starting structures as well as the Bergman cyclization without any substituents. All structures were ran at the CCSD/cc-pVDZ level of theory; with structure R being ran at restricted and all other structures being unrestricted. For comparison, the cyclization of (Z)-hexa-3-ene-1,5-diyne at the same level of theory is ΔE_a = 36.39 and ΔE_{rxn} = 7.84 kcal/mol.

Chalcogen (X= O, S, Se)	ΔE_a	ΔE_{rxn}
**Bergman cyclization**		
Oxygen	34.65	6.07
Sulfur	34.83	3.71
Selenium	33.98	3.47
**Chalcogen cyclization**		
Oxygen	7.72	-17.35
Sulfur	8.66	-10.54
Selenium	6.47	-13.66
**Bergman cyclization of chalcogen cyclized product**		
Oxygen	35.09	18.04
Sulfur	36.00	5.04
Selenium	35.97	4.61
**Chalcogen cyclization of Bergman cyclized product**		
Oxygen	6.79	-5.37
Sulfur	9.42	-9.20
Selenium	6.98	-12.52

a.

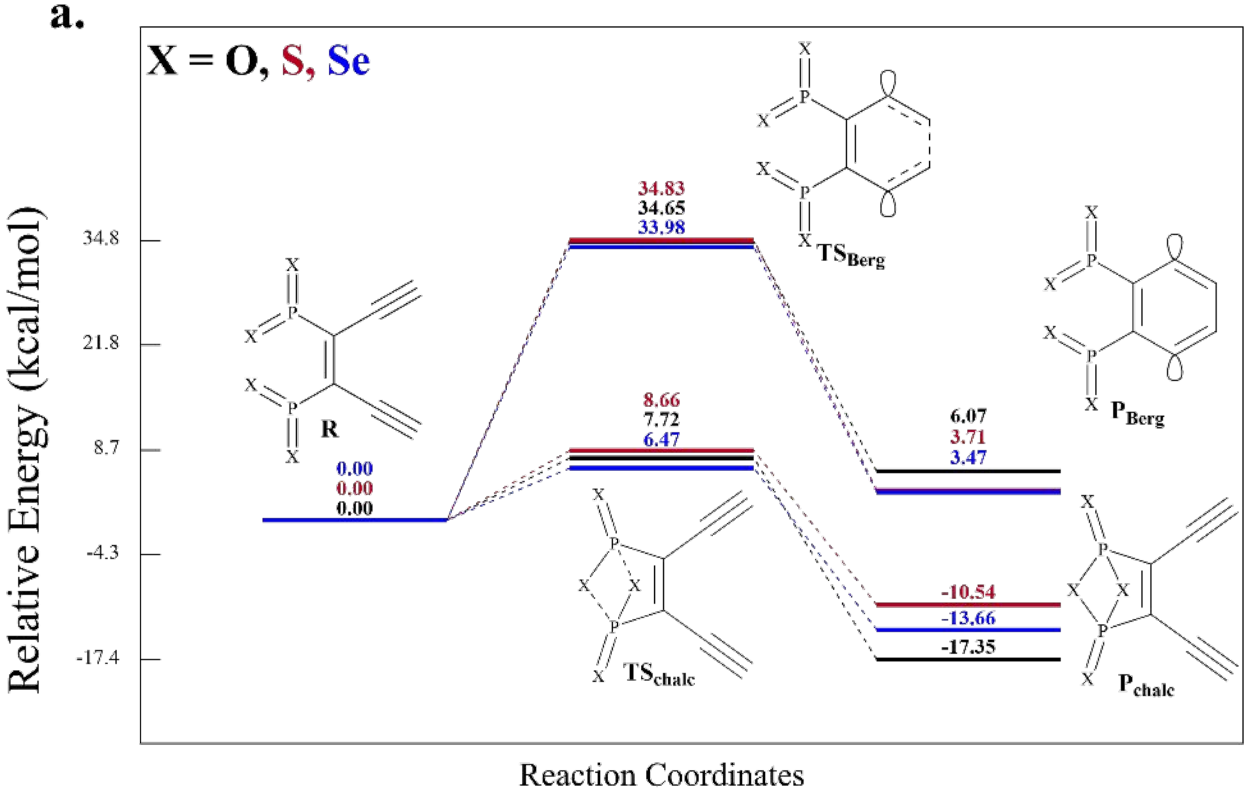
X = O, S, Se
Relative Energy (kcal/mol)
34.8
21.8
8.7
-4.3
-17.4
Reaction Coordinates
R
0.00
0.00
0.00
34.83
34.65
33.98
TS$_{Berg}$
8.66
7.72
6.47
TS$_{chalc}$
6.07
3.71
3.47
P$_{Berg}$
-10.54
-13.66
-17.35
P$_{chalc}$

b.

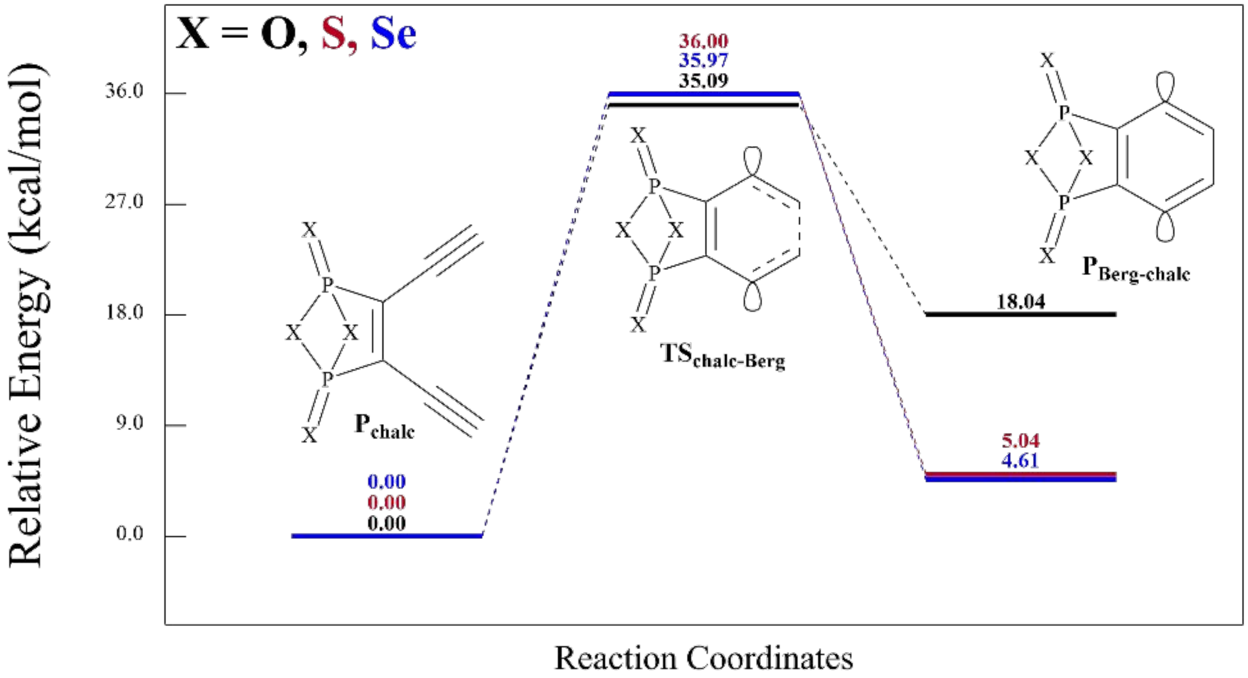
X = O, S, Se
Relative Energy (kcal/mol)
36.0
27.0
18.0
9.0
0.0
Reaction Coordinates
P$_{chalc}$
0.00
0.00
0.00
36.00
35.97
35.09
TS$_{chalc-Berg}$
18.04
5.04
4.61
P$_{Berg-chalc}$

c.

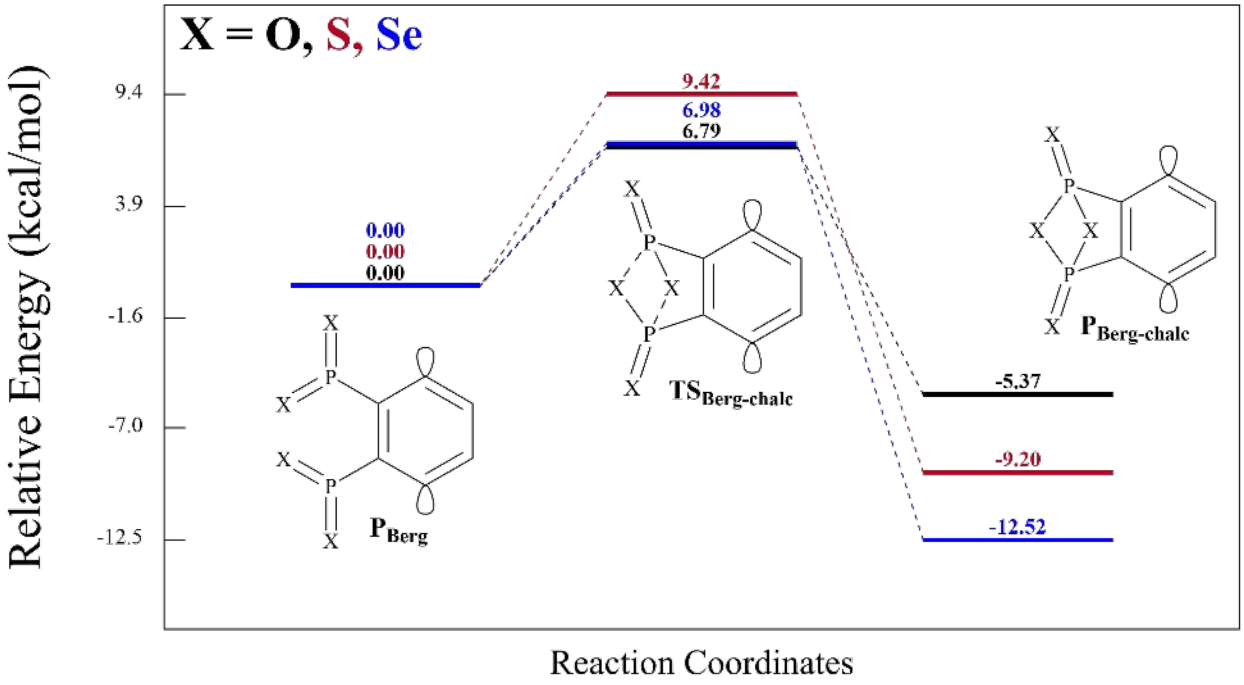
X = O, S, Se
Relative Energy (kcal/mol)
9.4
3.9
-1.6
-7.0
-12.5
9.42
6.98
6.79
0.00
0.00
0.00
-5.37
-9.20
-12.52
P_{Berg}
TS_{Berg-chalc}
P_{Berg-chalc}
Reaction Coordinates

d.

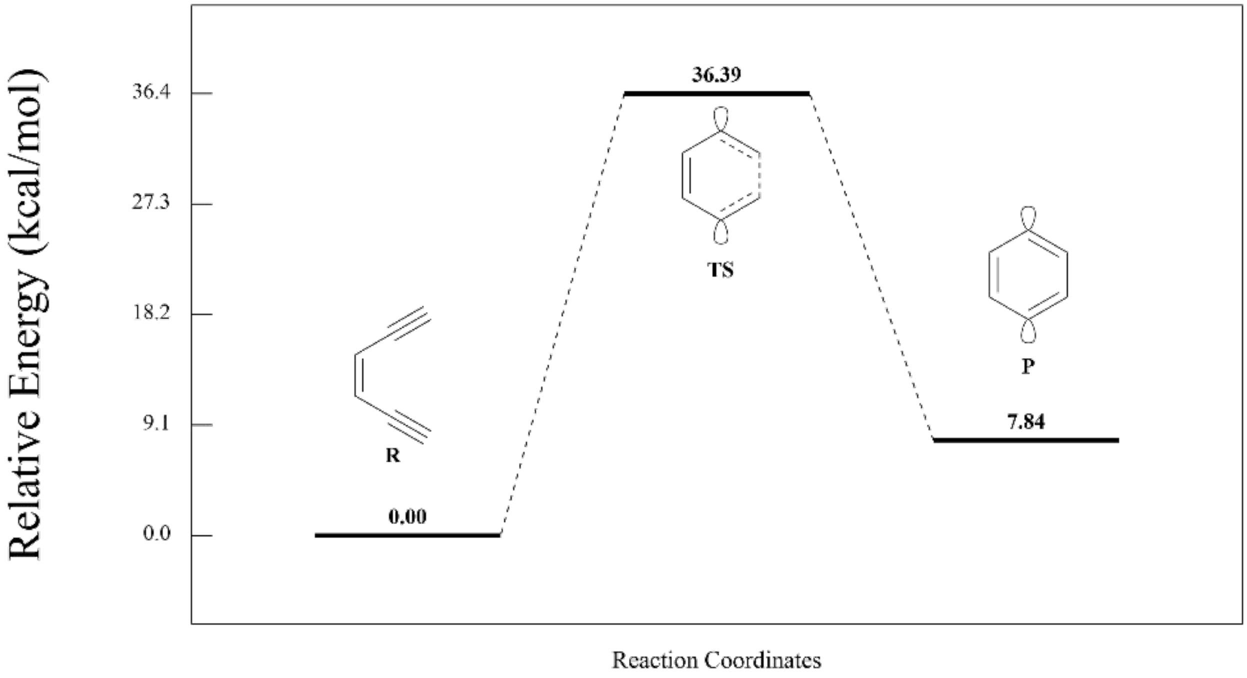
Relative Energy (kcal/mol)
36.4
27.3
18.2
9.1
0.0
36.39
7.84
0.00
R
TS
P
Reaction Coordinates

e.

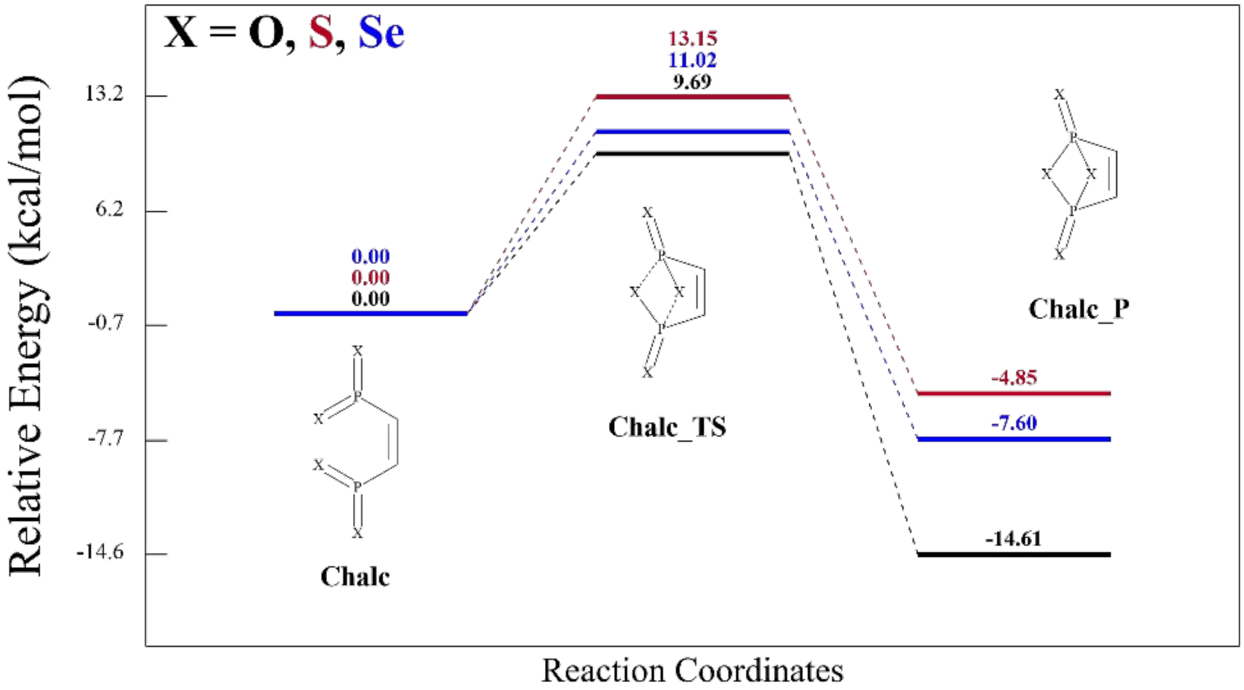

Figure S11 CCSD/cc-pVDZ potential energy diagram **(a)** of the Bergman cyclization and the chalcogen cyclization relative to **R**, **(b)** of the Bergman cyclization relative to $\mathbf{P_{chalc}}$, **(c)** of the chalcogen cyclization relative to $\mathbf{P_{Berg}}$, **(d)** for the Bergman cyclization of (Z)-hexa-3-ene-1,5-diyne, **(e)** for the chalcogen cyclization of (Z)-1,2-bis(dioxo/thio/seleno)phosphoranyl)ethene.

Table S11a. Leading determinants in the total ground state wavefunction | $X\ ^1A$ ⟩ for the oxygen congener of $\mathbf{P_{Berg}}$, constructed at the EOM-SF-UCCSD/cc-pVDZ level of theory from spin-flip excitation of high-spin triplet reference | 1 ^3A ⟩ prepared with UHF/cc-pVDZ orbitals.

Weight (%)[(a)]	Orbital Determinant[(b)]	Chemical Determinant[(c)]	2-Level[(d-f)]
Reference	$\vert$ (core)^{98} (43 a)$^{\alpha}$ (49 a)$^{\alpha}$ ⟩	$\vert$ (core)^{98} (1 σ*)$^{\alpha}$ (1 σ)$^{\alpha}$ ⟩	
47.2	$\vert$ (core)^{98} (43 a)$^{\alpha}$ (51 a)$^{\beta}$ ⟩	$\vert$ (core)^{98} (1 σ*)$^{\alpha\beta}$ ⟩	TCS #1
9.4	$\vert$ (core)^{98} (49 a)$^{\alpha}$ (52 a)$^{\beta}$ ⟩	$\vert$ (core)^{98} (1 σ)$^{\alpha\beta}$ ⟩	TCS #1
6.6	$\vert$ (core)^{96} (46 a)$^{\beta}$ (51 a)$^{\beta}$ (43 a)$^{\alpha}$ (49 a)$^{\alpha}$ ⟩	$\vert$ (core)^{98} (1 σ*)$^{\alpha\beta}$ (1 σ)$^{\alpha}$ ⟩	TCS #2
5.3	$\vert$ (core)^{98} (51 a)$^{\beta}$ (49 a)$^{\alpha}$ ⟩	$\vert$ (core)^{98} (1 σ*)$^{\beta}$ (1 σ)$^{\alpha}$ ⟩	OSS #1
4.3	$\vert$ (core)^{96} (42 a)$^{\beta}$ (51 a)$^{\beta}$ (43 a)$^{\alpha}$ (49 a)$^{\alpha}$ ⟩	$\vert$ (core)^{98} (1 σ*)$^{\alpha\beta}$ (1 σ)$^{\alpha}$ ⟩	TCS #3
3.1	$\vert$ (core)^{96} (49 a)$^{\alpha\beta}$ (54 a)$^{\alpha}$ (43 a)$^{\alpha}$ ⟩		
2.3	$\vert$ (core)^{96} (43 a)$^{\alpha\beta}$ (54 a)$^{\alpha}$ (49 a)$^{\alpha}$ ⟩		
1.3	$\vert$ (core)^{98} (52 a)$^{\beta}$ (43 a)$^{\alpha}$ ⟩	$\vert$ (core)^{98} (1 σ)$^{\beta}$ (1 σ*)$^{\alpha}$ ⟩	OSS #1
1.2	$\vert$ (core)^{96} (33 a)$^{\alpha}$ (51 a)$^{\beta}$ (43 a)$^{\alpha}$ (49 a)$^{\alpha}$ ⟩		
1.1	$\vert$ (core)^{96} (42 a)$^{\beta}$ (52 a)$^{\beta}$ (43 a)$^{\alpha}$ (49 a)$^{\alpha}$ ⟩	$\vert$ (core)^{98} (1 σ)$^{\alpha\beta}$ (1 σ*)$^{\alpha}$ ⟩	TCS #3

(a) Determinant weights computed as the square of EOM-SF-UCCSD excitation amplitudes
(b) “Singly occupied” molecular orbitals (i.e., those without a “partner” in the spin-flipped determinant relative to the original reference wavefunction) shown explicitly
(c) Representation of spin-flipped determinant with respect to singly occupy orbitals with a clear chemical interpretation (i.e., σ, σ)
(d) Contribution of spin-flipped determinants to a spin-complete two-configurational electronic state within the classic (2e, 2o) model for two radical electrons in two radical orbitals (σ & σ*). When multiple such states are present, they are numbered in decreasing order of weighted contribution to the total ground state wavefunction.
(e) TCS = “two-configurational closed-shell singlet”
(f) OSS = “open-shell singlet”

Table S11b. Leading determinants in the total ground state wavefunction | $X\ ^1A$ ⟩ for the sulfur congener of $\mathbf{P_{Berg}}$, constructed at the EOM-SF-UCCSD/cc-pVDZ level of theory from spin-flip excitation of high-spin triplet reference | 1 ^3B ⟩ prepared with UHF/cc-pVDZ orbitals.

**Weight (%)[(a)]**	**Orbital Determinant[(b)]**	**Chemical Determinant[(c)]**	**2-Level[(d-f)]**
Reference	$\lvert (\mathrm{core})^{130} (28\ b)^{\alpha} (32\ a)^{\alpha} \rangle$	$\lvert (\mathrm{core})^{130} (1\ \sigma^*)^{\alpha} (1\ \sigma)^{\alpha} \rangle$	
38.4	$\lvert (\mathrm{core})^{128} (28\ b)^{\alpha} (32\ a)^{\alpha} (30\ a)^{\beta} (34\ b)^{\beta} \rangle$	$\lvert (\mathrm{core})^{126} (1\ \sigma^*)^{\alpha\beta} (2\ \sigma^*)^{\alpha} (2\ \sigma)^{\alpha} (27\ b)^{\beta} (30\ a)^{\beta} \rangle$	TCS #1
12.3	$\lvert (\mathrm{core})^{128} (28\ b)^{\alpha} (32\ a)^{\alpha} (27\ b)^{\beta} (35\ a)^{\beta} \rangle$	$\lvert (\mathrm{core})^{126} (1\ \sigma)^{\alpha\beta} (2\ \sigma^*)^{\alpha} (2\ \sigma)^{\alpha} (27\ b)^{\beta} (30\ a)^{\beta} \rangle$	TCS #1
7.5	$\lvert (\mathrm{core})^{130} (28\ b)^{\alpha} (34\ b)^{\beta} \rangle$	$\lvert (\mathrm{core})^{128} (1\ \sigma^*)^{\alpha\beta} (2\ \sigma^*)^{\alpha} (2\ b)^{\beta} \rangle$	TCS #2
6.4	$\lvert (\mathrm{core})^{128} (30\ a)^{\alpha} (32\ a)^{\alpha} (30\ a)^{\beta} (35\ a)^{\beta} \rangle$		
3.7	$\lvert (\mathrm{core})^{128} (28\ b)^{\alpha} (32\ a)^{\alpha} (30\ a)^{\beta} (33\ b)^{\beta} \rangle$		
3.6	$\lvert (\mathrm{core})^{128} (28\ b)^{\alpha} (32\ a)^{\alpha} (27\ b)^{\beta} (36\ a)^{\beta} \rangle$		
2.1	$\lvert (\mathrm{core})^{128} (28\ b)^{\alpha} (32\ a)^{\alpha} (24\ a)^{\beta} (34\ b)^{\beta} \rangle$		
2.0	$\lvert (\mathrm{core})^{128} (28\ b)^{\alpha} (32\ a)^{\alpha} (27\ b)^{\beta} (37\ a)^{\beta} \rangle$	$\lvert (\mathrm{core})^{128} (2\ \sigma)^{\alpha\beta} (2\ \sigma^*)^{\alpha} (27\ b)^{\beta} \rangle$	TCS #2
1.8	$\lvert (\mathrm{core})^{128} (28\ b)^{\alpha} (32\ a)^{\alpha} (28\ b)^{\beta} (36\ a)^{\beta} \rangle$		
1.2	$\lvert (\mathrm{core})^{128} (28\ b)^{\alpha} (32\ a)^{\alpha} (32\ a)^{\beta} (34\ b)^{\beta} \rangle$		
1.1	$\lvert (\mathrm{core})^{128} (28\ b)^{\alpha} (32\ a)^{\alpha} (27\ a)^{\beta} (34\ b)^{\beta} \rangle$		

(a) Determinant weights computed as the square of EOM-SF-UCCSD excitation amplitudes
(b) "Singly occupied" molecular orbitals (i.e., those without a "partner" in the spin-flipped determinant relative to the original reference wavefunction) shown explicitly
(c) Representation of spin-flipped determinant with respect to singly occupy orbitals with a clear chemical interpretation (i.e., σ, σ*)
(d) Contribution of spin-flipped determinants to a spin-complete two-configurational electronic state within the classic (2*e*, 2*o*) model for two radical electrons in two radical orbitals (σ & σ*). When multiple such states are present, they are numbered in decreasing order of weighted contribution to the total ground state wavefunction.
(e) TCS = "two-configurational closed-shell singlet"
(f) OSS = "open-shell singlet"

Table S11c. Leading determinants in the total ground state wavefunction $|\, X\, ^1A\, \rangle$ for the selenium congener of $\mathbf{P_{Berg}}$, constructed at the EOM-SF-UCCSD/cc-pVDZ level of theory from spin-flip excitation of high-spin triplet reference $|\, 1\, ^3B\, \rangle$ prepared with UHF/cc-pVDZ orbitals.

**Weight (%)[(a)]**	**Orbital Determinant[(b)]**	**Chemical Determinant[(c)]**	**2-Level[(d-f)]**
Reference	$|\,(\text{core})^{202}\,(45\,b)^{\alpha}\,(48\,a)^{\alpha}\,\rangle$	$|\,(\text{core})^{202}\,(1\,\sigma^*)^{\alpha}\,(1\,\sigma)^{\alpha}\,\rangle$	
43.1	$|\,(\text{core})^{202}\,(45\,b)^{\alpha}\,(52\,b)^{\beta}\,\rangle$	$|\,(\text{core})^{202}\,(1\,\sigma^*)^{\alpha}\,(2\,\sigma^*)^{\beta})\,\rangle$	OSS #1
12.8	$|\,(\text{core})^{202}\,(48\,a)^{\alpha}\,(53\,a)^{\beta}\,\rangle$	$|\,(\text{core})^{202}\,(1\,\sigma)^{\alpha\beta}\,\rangle$	TCS #1
6.0	$|\,(\text{core})^{202}\,(48\,a)^{\alpha}\,(55\,a)^{\beta}\,\rangle$	$|\,(\text{core})^{202}\,(1\,\sigma)^{\alpha}\,(2\,\sigma)^{\beta}\,\rangle$	OSS #2
5.4	$|\,(\text{core})^{202}\,(48\,a)^{\alpha}\,(54\,a)^{\beta}\,\rangle$	$|\,(\text{core})^{202}\,(1\,\sigma)^{\alpha}\,(54\,a)^{\beta}\,\rangle$	OSS #3
3.3	$|\,(\text{core})^{200}\,(45\,b)^{\alpha}\,(48\,a)^{\alpha}\,(49\,a)^{\beta}\,(52\,b)^{\beta}\,\rangle$	$|\,(\text{core})^{200}\,(1\,\sigma^*)^{\alpha}\,(1\,\sigma)^{\alpha}\,(49\,a)^{\beta}\,(2\,\sigma^*)^{\beta}\,\rangle$	OSS #4
2.3	$|\,(\text{core})^{202}\,(45\,b)^{\alpha}\,(51\,b)^{\beta}\,\rangle$	$|\,(\text{core})^{202}\,(1\,\sigma^*)^{\alpha\beta}\,\rangle$	TCS #1
2.2	$|\,(\text{core})^{200}\,(45\,b)^{\alpha}\,(48\,a)^{\alpha}\,(42\,a)^{\beta}\,(52\,b)^{\beta}\,\rangle$	$|\,(\text{core})^{200}\,(1\,\sigma^*)^{\alpha}\,(1\,\sigma)^{\alpha}\,(42\,a)^{\beta}\,(2\,\sigma^*)^{\beta}\,\rangle$	OSS #5
1.5	$|\,(\text{core})^{200}\,(45\,b)^{\alpha}\,(48\,a)^{\alpha}\,(45\,a)^{\beta}\,(52\,b)^{\beta}\,\rangle$	$|\,(\text{core})^{200}\,(1\,\sigma^*)^{\alpha}\,(1\,\sigma)^{\alpha}\,(45\,a)^{\beta}\,(2\,\sigma^*)^{\beta}\,\rangle$	OSS #6
1.5	$|\,(\text{core})^{200}\,(45\,b)^{\alpha}\,(48\,a)^{\alpha}\,(46\,b)^{\beta}\,(53\,a)^{\beta}\,\rangle$	$|\,(\text{core})^{200}\,(1\,\sigma)^{\alpha\beta}\,(1\,\sigma^*)^{\alpha}\,(46\,b)^{\beta}\,\rangle$	TCS #2
1.4	$|\,(\text{core})^{200}\,(45\,b)^{\alpha}\,(48\,a)^{\alpha}\,(50\,a)^{\beta}\,(52\,b)^{\beta}\,\rangle$	$|\,(\text{core})^{200}\,(1\,\sigma^*)^{\alpha}\,(1\,\sigma)^{\alpha}\,(50\,a)^{\beta}\,(2\,\sigma^*)^{\beta}\,\rangle$	OSS #7

(a) Determinant weights computed as the square of EOM-SF-UCCSD excitation amplitudes
(b) "Singly occupied" molecular orbitals (i.e., those without a "partner" in the spin-flipped determinant relative to the original reference wavefunction) shown explicitly
(c) Representation of spin-flipped determinant with respect to singly occupy orbitals with a clear chemical interpretation (i.e., σ, σ*)
(d) Contribution of spin-flipped determinants to a spin-complete two-configurational electronic state within the classic (2*e*, 2*o*) model for two radical electrons in two radical orbitals (σ & σ*). When multiple such states are present, they are numbered in decreasing order of weighted contribution to the total ground state wavefunction.
(e) TCS = "two-configurational closed-shell singlet"
(f) OSS = "open-shell singlet"

Table S12a. Leading determinants in the total ground state wavefunction | $X\ ^1A_1$ ⟩ for the oxygen congener of $\mathbf{P}_{\mathbf{Berg\text{-}chalc}}$, constructed at the EOM-SF-UCCSD/cc-pVDZ level of theory from spin-flip excitation of high-spin triplet reference | 1 $^3B_2$ ⟩ prepared with UHF/cc-pVDZ orbitals.

**Weight (%)**[a]	**Orbital Determinant**[b]	**Chemical Determinant**[c]	**2-Level**[d-f]
Reference	$\vert\,(\text{core})^{98}\,(16\ b_2)^\alpha\,(22\ a_1)^\alpha\,\rangle$	$\vert\,(\text{core})^{96}\,(1\ \sigma^*)^\alpha\,(1\ \sigma)^\alpha\,\rangle$	
59.0	$\vert\,(\text{core})^{98}\,(16\ b_2)^\alpha\,(17\ b_2)^\beta\,\rangle$	$\vert\,(\text{core})^{96}\,(1\ \sigma^*)^{\alpha\beta}\,\rangle$	TCS #1
18.0	$\vert\,(\text{core})^{98}\,(22\ a_1)^\alpha\,(22\ a_1)^\beta\,\rangle$	$\vert\,(\text{core})^{96}\,(1\ \sigma)^{\alpha\beta}\,\rangle$	TCS #1
3.4	$\vert\,(\text{core})^{96}\,(16\ b_2)^\alpha\,(22\ a_1)^\alpha\,(21\ a_1)^\beta\,(17\ b_2)^\beta\,\rangle$		
2.4	$\vert\,(\text{core})^{96}\,(16\ a_1)^\beta\,(17\ b_2)^\beta\,(16\ b_2)^\alpha\,(22\ a_1)^\alpha\,\rangle$		
1.6	$\vert\,(\text{core})^{96}\,(16\ b_2)^\beta\,(23\ a_1)^\beta\,(16\ b_2)^\alpha\,(22\ a_1)^\alpha\,\rangle$		
1.4	$\vert\,(\text{core})^{96}\,(20\ a_1)^\beta\,(17\ b_2)^\beta\,(16\ b_2)^\alpha\,(22\ a_1)^\alpha\,\rangle$		
1.3	$\vert\,(\text{core})^{96}\,(15\ a_1)^\beta\,(17\ b_2)^\beta\,(16\ b_2)^\alpha\,(22\ a_1)^\alpha\,\rangle$		

(a) Determinant weights computed as the square of EOM-SF-UCCSD excitation amplitudes
(b) "Singly occupied" molecular orbitals (i.e., those without a "partner" in the spin-flipped determinant relative to the original reference wavefunction) shown explicitly
(c) Representation of spin-flipped determinant with respect to singly occupy orbitals with a clear chemical interpretation (i.e., σ, σ*)
(d) Contribution of spin-flipped determinants to a spin-complete two-configurational electronic state within the classic (2*e*, 2*o*) model for two radical electrons in two radical orbitals (σ & σ*). When multiple such states are present, they are numbered in decreasing order of weighted contribution to the total ground state wavefunction.
(e) TCS = "two-configurational closed-shell singlet"
(f) OSS = "open-shell singlet"

Table S12b. Leading determinants in the total ground state wavefunction | $X\ ^1A_1$ ⟩ for the sulfur congener of $\mathbf{P_{Berg\text{-}chalc}}$, constructed at the EOM-SF-UCCSD/cc-pVDZ level of theory from spin-flip excitation of high-spin triplet reference | 1 $^3B_2$ ⟩ prepared with UHF/cc-pVDZ orbitals.

Weight (%)[(a)]	Orbital Determinant[(b)]	Chemical Determinant[(c)]	2-Level[(d-f)]
Reference	$\vert (\text{core})^{130} (20\ b_2)^{\alpha} (28\ a_1)^{\alpha} \rangle$	$\vert (\text{core})^{130} (2\ \sigma^*)^{\alpha} (4\ \sigma)^{\alpha} \rangle$	
19.9	$\vert (\text{core})^{128} (20\ b_2)^{\alpha} (28\ a_1)^{\alpha} (26\ a_1)^{\beta} (22\ b_2)^{\beta} \rangle$	$\vert (\text{core})^{128} (2\ \sigma^*)^{\alpha\beta} (4\ \sigma)^{\alpha} (26\ a_1)^{\beta} \rangle$	TCS #1
19.1	$\vert (\text{core})^{128} (20\ b_2)^{\alpha} (28\ a_1)^{\alpha} (19\ b_2)^{\beta} (28\ a_1)^{\beta} \rangle$	$\vert (\text{core})^{126} (1\ \sigma)^{\alpha\beta} (2\ \sigma^*)^{\alpha} (4\ \sigma)^{\alpha} (25\ a_1)^{\beta} (19\ b_2)^{\beta} \rangle$	TCS #2
17.1	$\vert (\text{core})^{128} (20\ b_2)^{\alpha} (28\ a_1)^{\alpha} (27\ a_1)^{\beta} (22\ b_2)^{\beta} \rangle$	$\vert (\text{core})^{128} (2\ \sigma^*)^{\alpha\beta} (4\ \sigma)^{\alpha} (27\ a_1)^{\beta} \rangle$	TCS #3
7.6	$\vert (\text{core})^{130} (20\ b_2)^{\alpha} (22\ b_2)^{\beta} \rangle$	$\vert (\text{core})^{130} (2\ \sigma^*)^{\alpha\beta} \rangle$	TCS #4
4.9	$\vert (\text{core})^{128} (20\ b_2)^{\alpha} (28\ a_1)^{\alpha} (26\ a_1)^{\beta} (21\ b_2)^{\beta} \rangle$	$\vert (\text{core})^{126} (1\ \sigma^*)^{\alpha\beta} (2\ \sigma^*)^{\alpha} (4\ \sigma)^{\alpha} (19\ b_2)^{\beta} (26\ a_1)^{\beta} \rangle$	TCS #5
4.3	$\vert (\text{core})^{128} (20\ b_2)^{\alpha} (28\ a_1)^{\alpha} (27\ a_1)^{\beta} (21\ b_2)^{\beta} \rangle$	$\vert (\text{core})^{126} (1\ \sigma^*)^{\alpha\beta} (2\ \sigma^*)^{\alpha} (4\ \sigma)^{\alpha} (19\ b_2)^{\beta} (27\ a_1)^{\beta} \rangle$	TCS #6
3.4	$\vert (\text{core})^{128} (20\ b_2)^{\alpha} (28\ a_1)^{\alpha} (19\ b_2)^{\beta} (29\ a_1)^{\beta} \rangle$	$\vert (\text{core})^{126} (2\ \sigma)^{\alpha\beta} (2\ \sigma^*)^{\alpha} (4\ \sigma)^{\alpha} (19\ b_2)^{\beta} (26\ a_1)^{\beta} \rangle$	TCS #5
2.2	$\vert (\text{core})^{128} (20\ b_2)^{\alpha} (28\ a_1)^{\alpha} (21\ a_1)^{\beta} (22\ b_2)^{\beta} \rangle$	$\vert (\text{core})^{128} (2\ \sigma^*)^{\alpha\beta} (4\ \sigma)^{\alpha} (21\ a_1)^{\beta} \rangle$	TCS #7
2.0	$\vert (\text{core})^{130} (20\ b_2)^{\alpha} (21\ b_2)^{\beta} \rangle$	$\vert (\text{core})^{128} (1\ \sigma^*)^{\alpha\beta} (2\ \sigma^*)^{\alpha} (19\ b_2)^{\beta} \rangle$	TCS #8
1.9	$\vert (\text{core})^{128} (20\ b_2)^{\alpha} (28\ a_1)^{\alpha} (19\ b_2)^{\beta} (32\ a_1)^{\beta} \rangle$	$\vert (\text{core})^{128} (2\ \sigma^*)^{\alpha} (4\ \sigma)^{\alpha} (19\ b_2)^{\beta} (32\ a_1)^{\beta} \rangle$	TCS #9

(a) Determinant weights computed as the square of EOM-SF-UCCSD excitation amplitudes
(b) "Singly occupied" molecular orbitals (i.e., those without a "partner" in the spin-flipped determinant relative to the original reference wavefunction) shown explicitly
(c) Representation of spin-flipped determinant with respect to singly occupy orbitals with a clear chemical interpretation (i.e., σ, σ*)
(d) Contribution of spin-flipped determinants to a spin-complete two-configurational electronic state within the classic (2*e*, 2*o*) model for two radical electrons in two radical orbitals (σ & σ*). When multiple such states are present, they are numbered in decreasing order of weighted contribution to the total ground state wavefunction.
(e) TCS = "two-configurational closed-shell singlet"
(f) OSS = "open-shell singlet"

Table S12c. Leading determinants in the total ground state wavefunction | $X\ ^1A_1$ ⟩ for the selenium congener of $\mathbf{P_{Berg\text{-}chalc}}$, constructed at the EOM-SF-UCCSD/cc-pVDZ level of theory from spin-flip excitation of high-spin triplet reference | 1 $^3B_2$ ⟩ prepared with UHF/cc-pVDZ orbitals.

Weight (%)[(a)]	Orbital Determinant[(b)]	Chemical Determinant[(c)]	2-Level[(d-f)]
Reference	| (core)^{202} (29 b_2)^α (40 a_1)^α ⟩	| (core)^{202} (2 σ*)^α (4 σ)^α ⟩	
35.7	| (core)^{200} (29 b_2)^α (40 a_1)^α (38 a_1)^β (31 b_2)^β ⟩	| (core)^{200} (2 σ*)^{αβ} (4 σ)^α (38 a_1)^β ⟩	TCS #1
13.0	| (core)^{200} (29 b_2)^α (40 a_1)^α (39 a_1)^β (31 b_2)^β ⟩	| (core)^{200} (2 σ*)^{αβ} (4 σ)^α (39 a_1)^β ⟩	TCS #2
11.8	| (core)^{200} (29 b_2)^α (40 a_1)^α (28 b_2)^β (41 a_1)^β ⟩	| (core)^{198} (2 σ)^{αβ} (2 σ*)^α (4 σ)^α (28 b_2)^β (29 b_2)^β ⟩	TCS #3
10.5	| (core)^{200} (29 b_2)^α (40 a_1)^α (28 b_2)^β (40 a_1)^β ⟩	| (core)^{198} (1 σ)^{αβ} (2 σ*)^α (4 σ*)^α (28 b_2)^β (29 b_2)^β ⟩	TCS #4
2.8	| (core)^{202} (29 b_2)^α (31 b_2)^β ⟩	| (core)^{202} (2 σ*)^{αβ} ⟩	TCS #5
2.6	| (core)^{200} (29 b_2)^α (40 a_1)^α (33 a_1)^β (31 b_2)^β ⟩	| (core)^{200} (2 σ*)^{αβ} (4 σ)^α (33 a_1)^β ⟩	TCS #6
2.2	| (core)^{200} (29 b_2)^α (40 a_1)^α (28 b_2)^β (30 b_2)^β ⟩	| (core)^{200} (2 σ*)^α (4 σ)^α (28 b_2)^β (1 σ*)^β ⟩	OSS #1
1.6	| (core)^{200} (29 b_2)^α (40 a_1)^α (38 a_1)^β (30 b_2)^β ⟩	| (core)^{198} (1 σ*)^{αβ} (2 σ*)^α (4 σ)^α (28 b_2)^β (29 b_2)^β ⟩	TCS #3
1.3	| (core)^{200} (29 b_2)^α (40 a_1)^α (28 b_2)^β (43 a_1)^β ⟩	| (core)^{200} (2 σ*)^α (4 σ)^α (28 b_2)^β (43 a_1)^β ⟩	OSS #2
1.3	| (core)^{200} (29 b_2)^α (40 a_1)^α (37 a_1)^β (31 b_2)^β ⟩	| (core)^{200} (2 σ*)^{αβ} (4 σ)^α (37 a_1)^β ⟩	TCS #7
1.2	| (core)^{200} (29 b_2)^α (40 a_1)^α (36 a_1)^β (31 b_2)^β ⟩	| (core)^{200} (2 σ*)^{αβ} (4 σ)^α (36 a_1)^β ⟩	TCS #8

(a) Determinant weights computed as the square of EOM-SF-UCCSD excitation amplitudes
(b) "Singly occupied" molecular orbitals (i.e., those without a "partner" in the spin-flipped determinant relative to the original reference wavefunction) shown explicitly
(c) Representation of spin-flipped determinant with respect to singly occupy orbitals with a clear chemical interpretation (i.e., σ, σ*)
(d) Contribution of spin-flipped determinants to a spin-complete two-configurational electronic state within the classic (2*e*, 2*o*) model for two radical electrons in two radical orbitals (σ & σ*). When multiple such states are present, they are numbered in decreasing order of weighted contribution to the total ground state wavefunction.
(e) TCS = "two-configurational closed-shell singlet"
(f) OSS = "open-shell singlet"
